# Motif oriented high-resolution analysis of ChIP-seq data reveals the topological order of CTCF and cohesin proteins on DNA

**DOI:** 10.1186/s12864-016-2940-7

**Published:** 2016-08-15

**Authors:** Gergely Nagy, Erik Czipa, László Steiner, Tibor Nagy, Sándor Pongor, László Nagy, Endre Barta

**Affiliations:** 1 Department of Biochemistry and Molecular Biology, University of Debrecen, Debrecen, H-4032 Hungary; 2 UD-GenoMed Medical Genomic Technologies Research & Development Services Ltd., Nagyerdei krt. 98., Debrecen, H-4032 Hungary; 3 Agricultural Genomics and Bioinformatics Group, Agricultural Biotechnology Institute, NARIC, Gödöllő, H-2100 Hungary; 4 Faculty of Information Technology and Bionics, Pázmány Péter Catholic University Budapest H-1083, Gödöllő, Hungary; 5 MTA-DE Lendület Immunogenomics Research Group, University of Debrecen, Debrecen, H-4032 Hungary; 6 Present address: Wellcome Trust Sanger Institute, Wellcome Genome Campus, Hinxton, Cambridge, CB10 1SA UK

**Keywords:** CTCF, cohesin, ChIP-seq, DNA loop

## Abstract

**Background:**

ChIP-seq provides a wealth of information on the approximate location of DNA-binding proteins genome-wide. It is known that the targeted motifs in most cases can be found at the peak centers. A high resolution mapping of ChIP-seq peaks could in principle allow the fine mapping of the protein constituents within protein complexes, but the current ChIP-seq analysis pipelines do not target the basepair resolution strand specific mapping of peak summits.

**Results:**

The approach proposed here is based on i) locating regions that are bound by a sufficient number of proteins constituting a complex; ii) determining the position of the underlying motif using either a direct or a de novo motif search approach; and iii) determining the exact location of the peak summits with respect to the binding motif in a strand specific manner. We applied this method for analyzing the CTCF/cohesin complex, which holds together DNA loops. The relative positions of the constituents of the complex were determined with one-basepair estimated accuracy. Mapping the positions on a 3D model of DNA made it possible to deduce the approximate local topology of the complex that allowed us to predict how the CTCF/cohesin complex locks the DNA loops. As the positioning of the proteins was not compatible with previous models of loop closure, we proposed a plausible “double embrace” model in which the DNA loop is held together by two adjacent cohesin rings in such a way that the ring anchored by CTCF to one DNA duplex encircles the other DNA double helix and vice versa.

**Conclusions:**

A motif-centered, strand specific analysis of ChIP-seq data improves the accuracy of determining peak positions. If a genome contains a large number of binding sites for a given protein complex, such as transcription factor heterodimers or transcription factor/cofactor complexes, the relative position of the constituent proteins on the DNA can be established with an accuracy that allow one to deduce the local topology of the protein complex. The proposed high resolution mapping approach of ChIP-seq data is applicable for detecting the contact topology of DNA-binding protein complexes.

**Electronic supplementary material:**

The online version of this article (doi:10.1186/s12864-016-2940-7) contains supplementary material, which is available to authorized users.

## Background

In chromatin immunoprecipitation combined with sequencing (ChIP-seq), DNA fragments represented by sequence reads correspond to those regions that can be chemically cross-linked with a protein in question. In case of transcription factors (TFs), the term “peak” is generally used to denote a loosely defined region to which an elevated number of reads can be mapped compared to the background, while the peak summit shows the highest coverage of the region. Peak summits are known to more-or-less coincide with the corresponding DNA elements [1]. It is also known that due to possible protein-protein cross-linking events, components of a protein complex that are not directly involved in specific DNA binding can produce peaks that overlap with the peaks of TFs that anchor them to DNA [2].

The organization of interphase chromatin is mediated, among other mechanisms, by the dynamic formation of loop structures held together by cohesin, an evolutionarily conserved ring-like protein complex. The tripartite cohesin ring itself consists of RAD21, SMC1 and SMC3 proteins [3, 4] and is believed to anchor to DNA via STAG1/2 and CTCF [5–12]. CCCTC-binding factor (CTCF) is an 11-zinc finger protein, which binds to specific recognition sites (CTSs) on the DNA [13–15] that are supposed to serve as insulator elements [11, 16]. Parts of the cohesin complex are known in terms of atomic detail [17, 18], but most structural studies refer to cohesin being involved in sister-chromatid cohesion. The cohesin ring is large enough to embrace two sister chromatids, but this connection is believed to be topological rather than sequence specific, such as in the case of chromatin loop formation [3]. It is hypothesized that the cohesin ring is similar both in interphase and in metaphase [9, 11, 19], but the chain topology of loop closure is not known in sufficient detail. Current models disagree even on fundamental points such as the number of DNA duplexes enclosed within a cohesin ring [4, 8, 9, 11, 12, 19–23]. The position of the ring relative to the CTCF molecules is also uncertain. For example, models suggested in current studies [22, 24] consistently depict the ring in a distal position with respect to the loop and the anchoring CTCF molecules. However, as far as we are aware, there are no experimental data available that directly support this view.

A recent chromatin conformation capture (3C) based *in situ* Hi-C study pointed out that CTSs flanking the loops had a strand specific orientation, where generally the 5’ CTS was on the forward strand while the 3’ CTS was on the reverse strand [22]. This orientation specificity was further confirmed experimentally by the inversion of an anchoring CTS [25]. Earlier, we assigned the RXR-activated enhancers to induced genes in bone marrow derived macrophages through the use of regions bordered by active insulators that were bound both by CTCF and RAD21 [26]. In some selected examples, we also showed by 3C-sequencing that these flanking CTSs could anchor DNA loops. By further scrutinizing our ChIP-seq data, we observed that there was a characteristic shift between the co-localizing CTCF and RAD21 peaks. These observations encouraged us to look for general patterns in the positions of cohesin-related ChIP-seq peaks in the hope of discovering further information about how the CTCF/cohesin complex closes the DNA loops. Here we use a novel high-resolution analysis of ChIP-seq data to build an approximate model of CTCF/cohesin driven chromatin loop formation.

In this work we study the contact positions of the CTCF and cohesin complex proteins relative to the CTCF transcription factor binding site. We apply a high-resolution, motif-centered analysis on the available human and mouse CTCF and cohesin ChIP-seq datasets and find a characteristic shift pattern between the peaks of CTCF and the components of the cohesin complex. Based on this pattern as well as the known biochemical and structural data about the DNA/CTCF/cohesin complex, we propose a new “double-embrace” model for DNA loop closure.

## Results and discussion

### The summit-based high-resolution ChIP-seq analysis shows a characteristic shift pattern between the DNA contact points of CTCF, STAG1/2, RAD21 and SMC1/3 proteins

The high-resolution mapping approach proposed here seeks to extend the conventional analyses in two respects. Firstly we analyzed the fragment frequency distribution of the peaks and determined the most likely location of the genomic contact region of the DNA-protein interactions by using summit positions. Subsequently, we then represented the predicted contact points in terms of a genomic distance with respect to a reference point that we chose here as the center of the CTS. This had two important consequences: Since the CTS is a non-palindromic element, it has a strand specific orientation and thus the relative contact point positions can have both positive and negative values. Secondly, if we mapped the contact points of co-localizing DNA-binding proteins, we could then define an average distance (shift) between them. Underlying these considerations was the assumption that the fine positional shifts that may exist between the contact points of cohesin proteins (CTCF, RAD21, SMC1/3 and STAG1/2) may reflect the 3D position of the components within the complex. The genomic locations could thus be converted to approximate 3D distance constraints by projecting the shifts onto a 3D model of DNA.

The bottleneck of this analysis however is data quality. Researchers familiar with traditional ChIP-seq analysis are well aware of the quasi-chaotic uncertainty of peak positions. This is in part a natural consequence of the dynamic nature of DNA binding, which can be even more pronounced in the case of protein complexes. As a consequence, we needed a large number of co-occurring peaks (“co-peaks”) of distinct proteins from several cell types in order to derive peak shift values between the components of a protein complex. Preliminary experiments showed that we needed several hundred good quality (high coverage and well-resolved) co-peak data in order to determine a shift value within an accuracy of one base pair (Additional file 1: Figure S1). Producing such a large amount of ChIP-seq data can be a formidable task if a protein has few recognition sites within the genome. Fortunately, CTCF and cohesin have a large number of binding sites within the genome, and in addition, there are many ChIP-seq studies available in public databases. So in principle, the analysis could be carried out using the large body of data available on CTCF/cohesin. However there were a few conditions that had to be considered prior to conducting the analysis. First, we needed good quality ChIP-seq data for more than one cohesin protein that had been analyzed simultaneously in the same cell or tissue type (Additional file 2: Tables S1 and S2). Then, in order to decrease the number of non-relevant peaks, we selected those CTSs around which CTCF and at least one cohesin protein has been detected (see data collection). For this analysis we used the combination of an in-house developed computational pipeline [27] and custom-made scripts. Briefly, the analysis included the identification of CTCF/cohesin peaks, the building of a consensus CTS set and finally the determination of shifts between summits relative to the CTSs.

**Fig. 1:**
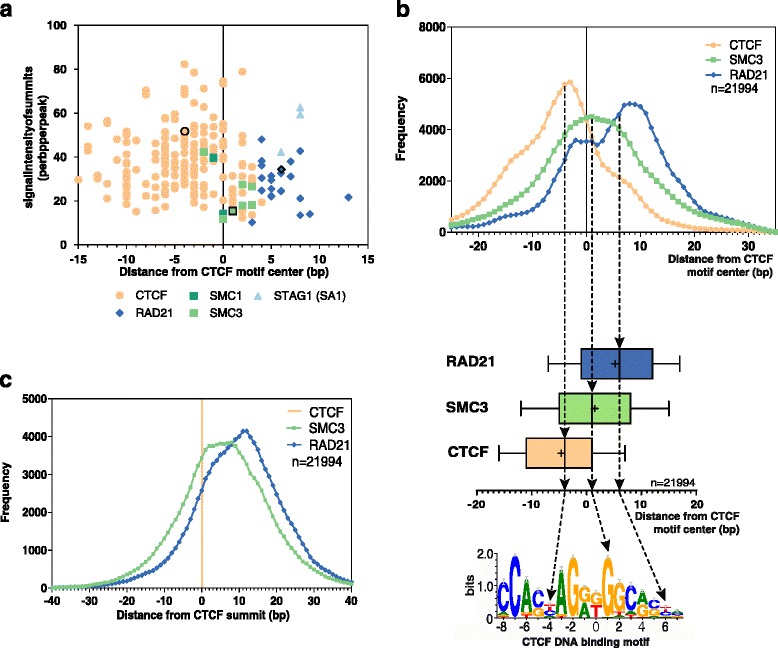
The shift between CTCF and cohesin bound sites. (**a**) The scatter plot shows the maxima of ChIP fragment coverage of CTCF, RAD21, SMC1/3 and STAG1 on CTSs specific for the given human cell or tissue type (Methods). The vertical axis shows the maxima of the average fragment depth and their position relative to the midpoint of CTSs is represented on the horizontal axis. HeLa results are marked with black border. (**b**) Distribution of the CTCF/cohesin proteins relative to the midpoint of 21,994 individual CTSs in HeLa cell line. Top: Histogram of summit distribution of the CTCF and cohesin bound sites using a 5 bp sliding window. Middle: Box plots indicate the mean (shown as ‘+’) and median (vertical line) peak summit positions of CTCF, SMC3 and RAD21 bound regions. The bottom panel shows the mapping on the CTCF motif logo (see Methods). (**c**) Distance distribution of cohesin proteins relative to the CTCF. Horizontal axis represents the distance of RAD21 (blue curve) and SMC3 summits (green curve) relative to the CTCF summits (orange line) and vertical axis represents the distance frequency. Rolling mean with 5 bp window was applied to smooth the frequency curves

The most critical part of this analysis was the filtering of raw data, which contained many CTCF sites around which all cohesin components could be found. In order to select a consistent high quality subset, we chose data “duos” and “trios” - i.e. regions in which one CTCF peak and a peak of at least one cohesin component were present within the same cell or tissue type (see data collection). Firstly, we selected 421 high quality human and mouse CTCF and cohesin ChIP-seq samples from public data repositories, and then strand-specifically determined the average summit positions relative to the center of the CTSs (Additional file 2: Tables S1 and S2). Even though the individual values showed a relatively broad dispersion, the analysis gave a surprisingly coherent picture: the serial order of peak summit positions was invariably CTCF – > SMC1/3 – > RAD21, STAG1/2 (Fig. 1; Additional file 1: Figures S2-S3) irrespective of whether the average positions were calculated for a cell type or for the entire dataset. Table S1 (Additional file 2) shows the tabulated values for the entire dataset (93 human cell types, 237 experiments) as well as for HeLa cells for which the most complete best quality data was available. Of the proteins studied, few ChIP-seq data were available for STAG1/2 in the public datasets, but it was clear that STAG proteins mapped to the 3’ end of the CTS, were overlapping with the RAD21 and were far from the CTCF positions (Fig. 1a; Additional file 1: Figure S2). Importantly, the shift patterns were highly conserved (*P* < 10^-15^ according to the Wilcoxon and Friedmann tests and *P* < 10^-9^ by simulation, see Additional file), even though some of the low quality datasets gave less significant results (Additional file 1: Table S4 and S5). We have also re-analyzed the available HeLa DNase-seq and CTCF ChIP-exo datasets and found that they exactly mark the borders of the region we had found to be occupied on the DNA by CTCF/cohesin proteins (Additional file 1: Figure S3). The same overall patterns were found for both human and mouse data (see Additional file).

Since CTCF is the only known specific DNA binder among the components of the CTCF/cohesin complex, we expected that the corresponding ChIP-seq peaks will point to the same position with respect to CTS. In contrast, the fact that we found conserved shift values suggests that also the SMC proteins, STAG1/2 and RAD21 occupy conserved – relatively fixed – positions that are close enough to DNA so as to give rise to DNA-protein crosslinks during the ChIP-seq procedure.

### The new “double-embrace” model explains both the biochemical and the shift pattern data

**Fig. 2:**
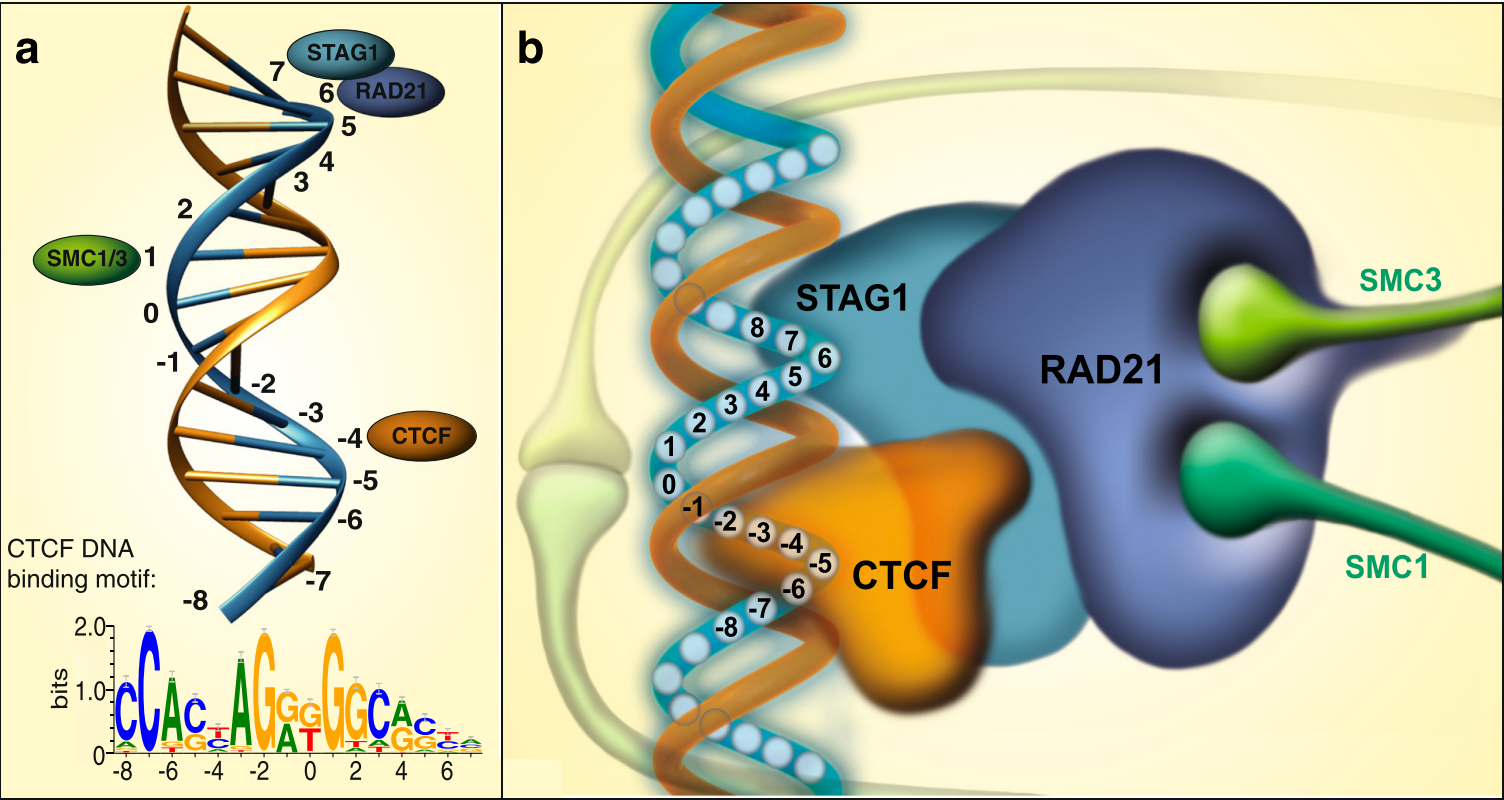
Transforming the shift values into the B-DNA. (**a**) Mapping the peak shift values of the cohesin components onto a schematic circle diagram of B-DNA (representing the top view of the helix) shows that CTCF, RAD21 and STAG1/2 map on one face of the helix while SMC1 and SMC3 map on the other face. (**b**) 3D arrangement of CTCF/cohesin complex on the DNA helix. The binding positions correspond to the median values indicated in Fig. 1

The next step was to convert the positional distances (shifts) into approximate 3D spatial constraints. For this, we chose the median positions from HeLa cells for CTCF (-4), SMC3 (+1) and RAD21 (+6), and the median position from MCF-7 cells for STAG1 (+7). This shift pattern was then mapped on the surface of a B-DNA model that we built using a sequence dependent modeling procedure [28]. The model building indicated that the chosen CTS was not inherently curved (Additional file 1: Figure S10). However, the putative contact positions of the individual proteins did map on opposite faces of the B-DNA double helix (Fig. 2a; Additional file 1: Figure S11). In more detail, the peak summits of CTCF, STAG1/2 and RAD21 mapped on one face of the double helix, while the contact sites of SMC1/3 were on the opposite face. It is interesting to note that it is the very same SMC1/3 positions that show the greatest discrepancy between the observed shift pattern and the known binding order of the cohesin proteins. Namely, the binding order of the contributing proteins according to current knowledge is DNA, followed by CTCF, STAG1/2, RAD21 and SMC1/3 [5, 7, 17–19], and yet the observed shift pattern was CTCF followed by SMC1/3, and RAD21/ STAG1/2 together. Our results clearly suggest that RAD21 and STAG are in contact not only with each other [17] but either one, or both of the proteins are in close contact with DNA, around the 3’ end of CTS. In such a manner, the relative position of RAD21/STAG with respect to CTCF can be clearly defined which is in agreement with the known CTCF/STAG interaction [7]. Importantly, Gligoris et al. showed that SMC3/RAD21/SMC1 is a compact structure in which SMC1 and SMC3 heads are in contact [18], which makes it unlikely that a large molecular complex such as the DNA/CTCF/STAG/RAD21 subcomplex could fit within the same ring. Somewhat counter-intuitively, the most plausible explanation for this discrepancy is that the SMC1/SMC3 molecules that generated this ChIP-seq signal possibly belong to another cohesin ring – that is to say the one linked to the opposite end of the DNA loop (Fig. 2b). Clearly, SMC1 and SMC3 are elongated, chain-like molecules that would form complicated, entangled loops when recoiling to the same helix that harbors their DNA anchor, CTCF (not shown). So, while a variety of ring topologies can be conceived, the most parsimonious model is to suppose that the cohesin ring anchored by CTCF to one DNA duplex embraces the other double helix between the CTCF and RAD21/STAG1/2 molecules and vice versa. We termed this mode of binding as the “double embrace” model, in order to distinguish it from the earlier ring [4, 17] and handcuff models [29] suggested for sister-chromatid binding and the model drawn recently by Sanborn et al for DNA loops [30]. The distinctive feature of the double embrace arrangement is that it explicitly includes the sequence specific link between cohesin and DNA as well as the arrangement of cohesin components with respect to the CTCF binding site.

Although this model was derived from an observed peak shift pattern (Fig. 1a), it is in agreement both with the loop-closing function of the complex and with the subunit interactions suggested in previous studies [5, 7, 17–19]. At the same time it suggests novel subunit interactions that can be experimentally tested, for instance the CTCF/RAD21 interaction that follows from the shift pattern. In particular, our model supports the recent findings of Rao et al. [22] and Guo et al. [25] who showed that the two CTS anchor sites flanking a DNA loop must align in a convergent orientation. At the same time, our data also answer the important question of Bouwman and de Laat regarding the position of cohesin ring(s) with respect to CTSs [12]. Namely, we found that the cohesin ring overlaps with the CTSs so that its center is slightly shifted towards the interior region of the loop.

The double embrace arrangement provides testable hypotheses that may help to clarify several, seemingly contradictory features of loop closure. Firstly, the loop has to be mechanically stable so as to fix the DNA molecule during transcription events. On the contrary, the loop has to be flexible so as to find its precise location on the DNA duplex. While the presence of two cohesin rings seemingly satisfies the stability criterion, the large number of intermolecular contacts of the double embrace structure may seem to contradict the need for flexibility. And yet, a sequential closure of the two rings might explain how a stable lock can form at a precise location of the DNA duplex. Namely, the ring formed first might glide along the DNA duplex and stop at a location where the SMC arms of the second ring lock the double ring structure. Such a scenario might in principle be deduced from a pattern of secondary peaks but the resolution of the current data does not allow this conclusion (data not shown). This semi-fixed or free gliding is also in accordance with the loop extrusion model in which cohesin ring(s) are moving along the loop until finding the anchor points [30].

Secondly, there is evidence that the hinge domain has DNA binding capability, and its opening and closing requires ATP-ase activity of the SMC head domain in both cohesin and condensin [31–33]. The hinge and head domains are separated by a relatively long rod like structure (approximately 45 nm in length), which in principle, should not favor interaction. In the double embrace structure, the SMC hinge domain of one ring is likely to be located in the vicinity of the head domain of the other ring, meaning that their apparent mean distance is only going to be a few nanometers, which may allow dynamic interactions. This effect only appears to happen when both rings are in the position of loop closure, with the consequence of the enzymatic reaction occurring only at the right place and the right time.

Third, the DNA duplex is known to form multiple loop structures [34, 35]. The double embrace model provides two clues regarding how this might happen. On the one hand, the double embrace can be easily extended to three (or more) DNA duplexes. Namely, in the double embrace structure, the ring anchored to duplex A encircles duplex B and vice versa. In a three duplex model, the ring anchored by CTCF to A encircles duplex B. The ring anchored to duplex B encircles duplex C, and the ring anchored to duplex C encircles duplex A. In other words, a triagonal structure can form in which the loops are connected by CTCF bound to a single cohesin ring. On the other hand, further loops can also form within a primary loop locked by a double embrace structure. In this structure, RAD21 and STAG1/2 proteins are facing the primary loop so they can interact with proteins bound to various sites within the original loop, forming multiple loop structures via protein/protein interactions.

### The summit-based high-resolution ChIP-seq analysis can be applied to mapping other transcription factor complexes

**Fig. 3:**
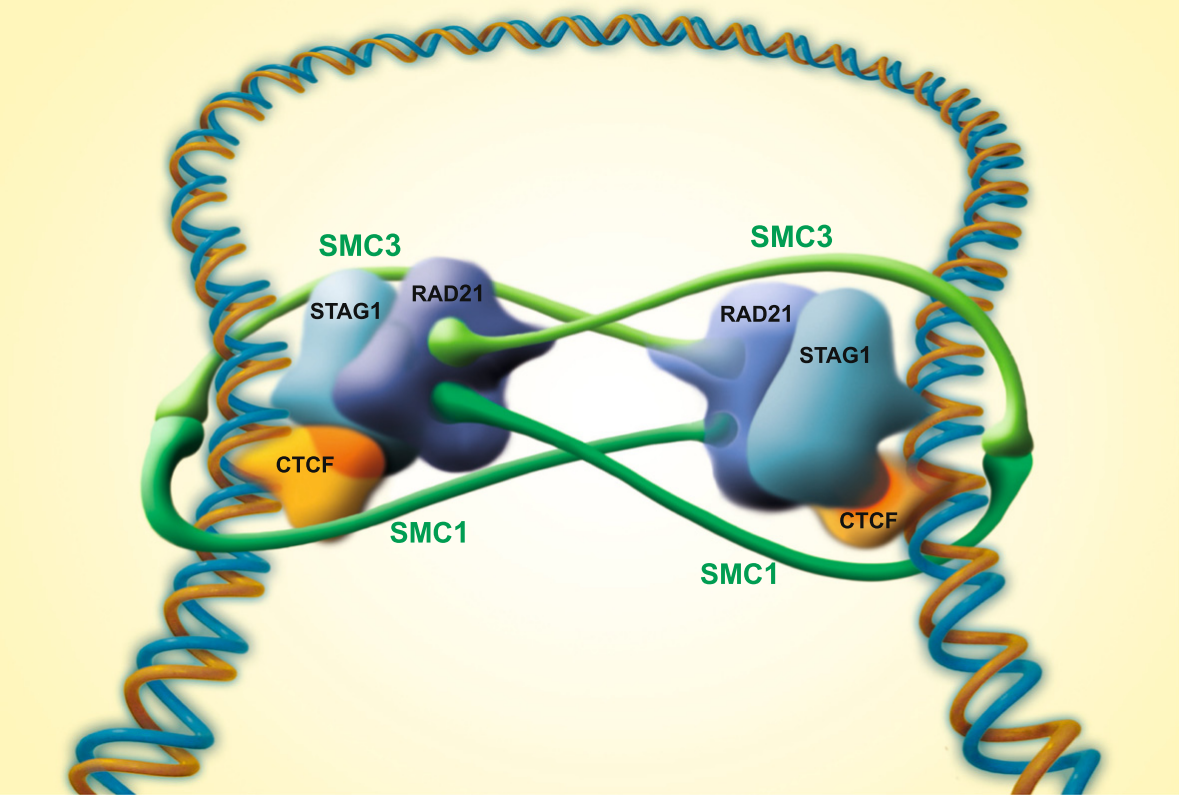
The “double embrace” model of DNA loop closure. The topology of the cohesin ring is derived from the positional values (Fig. 1) and the 3D arrangement (Fig. 2). The model explains how a DNA-loop is fixed by flanking CTCF/cohesin complexes

As far as the approach is concerned, the high-resolution, motif-specific analysis of ChIP-seq data described here can be applied to the analysis of other biologically relevant complexes. For instance it allows one to determine the spatial orientation of a protein binding to a DNA-bound transcription factor. If the binding is symmetrical, the ChIP-seq peaks will center around the same average position. If the binding is asymmetrical, there will be a shift in the positions. This will occur whenever the protein binds to a site on DNA, which is vicinal to the transcription factor binding site, and also, if the protein is part of a larger complex that stabilizes it in an asymmetrical position. In our case, the binding regions of CTCF and RAD21 were found to be vicinal but not overlapping. Due to the fact that RAD21 is not a specific DNA binder, we conclude that it has to be part of an asymmetrical complex, and that helped us to formulate the double embrace model shown in Fig. 3. In Additional file we present a case study (Additional file 1: Figure S12), in which an asymmetric binding complex (the FOXA1/AR) showed a highly significant 4 bp shift between its components (*P* < 2.2x10^-16^ according to the Wilcoxon signed-rank test), while symmetrically binding controls show no shift (Additional file 1: Figure S13), which gives further support to the general applicability of the analysis principle.

## Conclusions

In summary, the 3D organization of the chromatin and its role in global regulation of gene expression is one of the most important but still poorly understood mechanisms in molecular biology. Our results should therefore go some distance towards clarifying this issue. Through the meta-analyses of cistromic datasets we could show that the cohesin ring is in proximal position at the DNA loops. We are proposing a double embrace model that involves two cohesin rings that can now help explain the formation and the dynamic nature of these chromatin loops. Our model can also help to determine the structure of the DNA/CTCF/cohesin complexes at a higher resolution and should now help understanding of the molecular processes occurring during the closing and opening of the cohesin rings. Finally, the high resolution ChIP-seq analysis that we introduced here offers a novel way to better visualize the spatial organization of DNA bound protein complexes.

## Methods

### Datasets

Human and mouse ChIP-seq, ChIA-PET, ChIP-exo and DNase-seq data were downloaded from the NCBI Sequence Read Archive [[36], http://www.ncbi.nlm.nih.gov/sra, 09.30.2015.] and the Encyclopedia of DNA Elements (ENCODE) [[37], http://genome.ucsc.edu/ENCODE/downloads.html, 09.30.2015.]. CTCF and cohesin (RAD21, SMC1/3 and STAG1) ChIP-seq datasets of each cell or tissue type (Additional file 2: Tables S1 and S2) were selected using the following intuitive criteria:

i) In the cases of CTCF and cohesin samples with common origin, the measurements were carried out under identical conditions.
ii) Sequencing was carried out on Illumina platform.
iii) The number of mapped reads was above 10 million. NCBI Build 37/hg19 and NCBI Build 38/mm10 were used as human and mouse reference genomes, respectively.

### Raw data processing

Processing of raw data (including short read mapping, peak calling, the finding of enriched motifs and the creation of genome browser compatible files for data visualization) was carried out with an in-house developed ChIP-seq analysis pipeline [27] using the steps listed in Table S3 (Additional file 1). [38–41]. For peak calling and raw peak summit determination we used MACS2 [39] and artifacts – based on the blacklisted genomic regions of ENCODE – were removed by intersectBed (BEDtools) [42].

### Determination of the consensus binding sites of CTCF

As we expected a single shift or no shift between the CTCF and the cohesin complex, we differentiated these two groups of proteins. As the average resolution of the ChIP-seq coverage is about a few tens of base pairs, we calculated the average position of the sites bound by CTCF (consensus peak summits) based on the raw peak summits if these were present in at least two samples and were closer than 51 bps. Consensus peak summits for cohesin were determined in the same way. Finally, we collected those consensus CTCF peak summits that were closer to a consensus cohesin summit than 51 bps. Direction of the shift between the consensus peak summits of CTCF and cohesin was determined and showed that the cohesin is almost always downstream compared to the CTCF protein on the DNA.

Motif enrichments were determined by findMotifsGenome.pl [40] from the 100 bp regions of the most ubiquitous 5000 CTCF/cohesin bound sites, in two rounds. In the second motif enrichment search we used the top 5000 regions lacking the CTCF element (CTS) hits of the first search (which were mapped by annotatePeaks.pl, Homer). Score 6 was set for both CTCF motif matrices to ensure that as many CTSs were located as possible. The mapped CTSs were then filtered. ~90 % of the motifs were alone on the co-peaks and 76.8 % of them followed the shift both in human and mouse. In the case of overlaps, we chose those hits having the highest motif score. In the case of multiple elements under a co-peak, we chose that putative element following the shift and having the highest motif score. In the case of multiple elements in the opposite direction, we also selected the one with the highest motif score.

### Determination of shifts between CTCF and cohesin bound sites

ChIP-fragment coverage of CTCF/cohesin samples were plotted on their own CTS set (where peaks of the individual sample overlap with the consensus CTS set) by annotatePeaks.pl using -hist 1 parameter [40]. The maxima of each histogram and the position of these values relative to the CTS center are shown as scatter plots on Fig. 1a and Additional file 1: Figure S2 for human and mouse cells, respectively.

To further investigate the strand specific shift between CTCF and cohesin peak summits, we compared sample duos and trios that were derived from the same cell or tissue type with identical condition. In these cases, for each comparison, peaks were selected that overlapped with the consensus CTS set. Instead of the summit predictions of MACS2, we used the ones located with PeakSplitter, which was developed to discriminate subpeaks (in case of overlapping peaks) and thus gives more accurate local maxima. The distance of the summits relative to the reference points was established by closestBed (BEDtools) [42]. Firstly, the reference point was the mathematical middle of the CTSs in the histograms and box plots showing the summit distribution of the CTCF and cohesin bound (Fig. 1b; Additional file 1: Figures S3B and S4-S7). Then, we set the CTCF summits as reference points (Fig. 1c; Additional file 1: Figures S3C and S8-S9).

### Investigation of CTCF/cohesin co-occupied sites with ChIP-exo and DNase-seq data

To identify the genomic location and coverage of CTCF/cohesin proteins with near-single-nucleotide accuracy, we used publicly available HeLa DNase-seq and ChIP-exo data (SRX100899, SRX098243).

The single-nucleotide resolution border peak detection was executed with “model based analysis of ChIP-exo” (MACE) [43].

The DNase-seq bam files were downloaded directly from the ENCODE database [37]. The raw sequence reads were then aligned to the hg19 human genome. The accurate prediction of CTCF/cohesin footprints were then done with the Wellington algorithm [44]. This algorithm detects characteristic depletions of DNase I cuts and compares the result with a large number of cuts in the surrounding region of open chromatin that do not harbor bound proteins.

The identified ChIP-exo and DNase-seq borders were then compared with the processed ChIP-seq data and are shown in Additional file 1: Figures S3B and S3C.

### Identification of CTSs involved in chromatin looping

ChIA-PET (Chromatin Interaction Analysis by Paired-End Tag Sequencing) data was used to collect CTSs that are involved in CTCF mediated chromatin looping. The CTCF ChIA-PET data were downloaded from the public database of ENCODE as a processed interaction set in “junction BED” format [37]. We used the interaction sets of the MCF7 cell line in further analyses because it has biological replicates and good quality RAD21 (SRX190247) and CTCF (SRX190190) ChIP-seq data that are also derived from the ENCODE database.

We identified CTSs under RAD21 and CTCF co-occupied region [42] with the findMotifsGenome.pl and annotatePeaks.pl analyses of ChIP-seq data [40]. The CTSs were used in the following analyses.

The two sides of one interaction are actually two sections of the DNA (ChIA-PET DNA section with variable size). The ChIA-PET DNA sections contain CTSs, which are involved in CTCF-mediated DNA looping. To identify these we searched for the closest CTS to the midpoint of the ChIA-PET DNA sections that showed convergent motif orientation with the CTS of the other side if there was interaction [42].

We constructed a consensus interaction set from the replicas using intersectBed [42]. 8482 interactions were selected and these showed 100 % overlap on both CTSs of the interaction (intersectBed -f 1 -r). A representative examples of the loops are shown in (Additional file 1: Figure S3A and Additional file 3: Table S11).

## Abbreviations

3C, Chromosome conformation capture; AR, androgen receptor; Bp, base pair; ChIA-PET, Chromatin Interaction Analysis with Paired-End-Tag sequencing; ChIP-exo, chomatin immunoprecipitation with exonuclease digestion and high-throughput sequencing; ChIP-seq, chromatin immunoprecipitation followed by sequencing; CTCF, CCCTC-binding factor; CTS, CTCF specific recognition sites; DHS, DNase I hypersensitivity sites; DNase-seq, deoxyribonuclease I digestion with high-throughput sequencing; ENCODE, ENCyclopedia Of DNA Elements; kb, kilobase; FOXA1, Forkhead box protein A1; MACE, model based analysis of ChIP-exo; MACS, Model-based Analysis of ChIP-Seq data; PET, paired-end tag; RAD21, Double-strand-break repair protein rad21 homolog; SMC, Structural Maintenance of Chromosomes; SMC1/3, constitutes the core subunits of the cohesin complexes involved in sister chromatid cohesion; STAG2, Cohesin subunit SA-2; TF, transcription factor; TFBS, transcription factor binding site

## Additional files

Additional file 1: Supplementary Materials. **Figure S1**. The reproducibility of ChIP-seq peak shifts in a HeLa cell experiment. **Figure S2**. Shift between CTCF and cohesin bound sites in mouse cells. **Figure S3**. The boundaries of genomic regions covered by CTCF/cohesin. **Figure S4**. Shift between CTCF/cohesin proteins in human cell lines. **Figure S5**. Box plot representation of the strand specific shift between CTCF and cohesin proteins in human cell lines. **Figure S6**. Shift between CTCF/cohesin proteins in mouse cell and tissue types. **Figure S7**. Box plot representation of the strand specific shift between CTCF and cohesin proteins in mouse cell and tissue types. **Figure S8**. Distance distribution of cohesin proteins relative to the CTCF in human cell lines. **Figure S9**. Distance distribution of cohesin proteins relative to the CTCF in mouse cell and tissue types. **Figure S10**. DNA modeling. The model of the CTCF binding site (CTS) and a consensus prediction of 16964 aligned binding sites shows that the DNA double helix is not inherently curved in this region (inset), and that it is slightly less curved and more flexible than the surrounding regions. **Figure S11**. Mapping the shift values onto the B-DNA. **Figure S12**. Shift between interacting transcription factors (positive control). **Figure S13**. Lack of shift between interacting transcriptional regulator proteins (negative control). **Table S3**. Steps of ChIP-seq analysis pipeline. **Table S4**. Results of statistic analysis in case of two coherent samples. **Table S5**. Results of statistic analysis in case of more then two coherent samples. **Table S6**. Summary table of CTCF-cohesin samples. **Table S7**. Average values of CTCF/cohesin proteins related to CTS. **Table S8**. Median and Mean distance from CTCF summits. **Table S9**. Standard deviation of protein distances near CTSs. **Table S10**. Relative positions of the co-occupied transcription regulators used as controls. (DOCX 1860 kb)

Additional file 2: **Table S1 and S2**. Basic statistics (and availability, SRA numbers) of human and mouse datasets, respectively. (XLSX 58 kb)

Additional file 3: **Table S11**. Investigation of curvature/bendability on CTS-centered sequences. The table shows the sequence of 400 bp frame of 16964 CTSs, which were identified with ChIA-PET analysis sequences. (XLSX 1305 kb)
