# Supplementary material for: Motif oriented high-resolution analysis of ChIP-seq data reveals the topological order of CTCF and cohesin proteins on DNA
Source: BMC Genomics. 2016 Aug 15;17:637. doi: 10.1186/s12864-016-2940-7 (PMC4986361; doi:10.1186/s12864-016-2940-7)
Supplement: Supplementary file 1 — Supplementary Materials. Figure S1. The reproducibility of ChIP-seq peak shifts in a HeLa cell experiment. Figure S2. Shift between CTCF and cohesin bound sites in mouse cells. Figure S3. The boundaries of genomic regions covered by CTCF/cohesin. Figure S4. Shift between CTCF/cohesin proteins in human cell lines. Figure S5. Box plot representation of the strand specific shift between CTCF and cohesin proteins in human cell lines. Figure S6. Shift between CTCF/cohesin proteins in mouse cell and tissue types. Figure S7. Box plot representation of the strand specific shift between CTCF and cohesin proteins in mouse cell and tissue types. Figure S8. Distance distribution of cohesin proteins relative to the CTCF in human cell lines. Figure S9. Distance distribution of cohesin proteins relative to the CTCF in mouse cell and tissue types. Figure S10. DNA modeling. The model of the CTCF binding site (CTS) and a consensus prediction of 16964 aligned binding sites shows that the DNA double helix is not inherently curved in this region (inset), and that it is slightly less curved and more flexible than the surrounding regions. Figure S11. Mapping the shift values onto the B-DNA. Figure S12. Shift between interacting transcription factors (positive control). Figure S13. Lack of shift between interacting transcriptional regulator proteins (negative control). Table S3. Steps of ChIP-seq analysis pipeline. Table S4. Results of statistic analysis in case of two coherent samples. Table S5. Results of statistic analysis in case of more then two coherent samples. Table S6. Summary table of CTCF-cohesin samples. Table S7. Average values of CTCF/cohesin proteins related to CTS. Table S8. Median and Mean distance from CTCF summits. Table S9. Standard deviation of protein distances near CTSs. Table S10. Relative positions of the co-occupied transcription regulators used as controls. (DOCX 1860 kb) [file 12864_2016_2940_MOESM1_ESM.docx]

Supplementary Materials:

**Data visualization and statistics**

Statistical calculations were carried out in the R environment (<http://www.R-project.org>) [1] using R version 3.1.2. Statistics were calculated using reshape2 and PMCMR [2, 3]. Matched samples were analyzed with the Wilcoxon signed-rank test (a two related sample comparison) and Friedmann test with Namenyi post-hoc test (to account for multiple test attempts). The threshold for significance was P<0.05.

Wilcoxon and Friedmann tests were made on the relative orientation of CTCF and cohesin proteins (Additional file 2: Tables S1 and S2). The results showed high significance (Tables S4 and S5).

To confirm this result we performed computer simulations to understand this more fully. Repeating the simulation 10 million times resulted in none of the attempts showing similar patterns to those that were experimentally observed. The source code of the program is available on <https://github.com/TravisCG/CTCFSim>.

Genome browser compatible files were made using BEDTools and makeUCSCfile [4, 5]. We used Integrative Genomics Viewer (IGV) in the data visualization phases [6].

The reproducibility of peak shift values was tested on the HeLa dataset. The peak shift was measured between the peak summit of the proteins indicated (CTCF, Rad21, SMC3) and the center of the CTCF binding site (CTS). In Figure S1, the reproducibility was characterized with the standard deviation of the mean (Y axis) that was determined from a number of observed peak shifts (X-axis). The inset shows a detail in logarithmic scale that shows that approximately 100 shift values are necessary to reach a reproducibility of +/-1 nucleotide. In our experiments we normally used more than 5000 peaks that roughly corresponded to a reproducibility of 0.1. Naturally the reproducibility estimates vary with the quality/coverage of the dataset. In practice, we rounded the peak shift values to one nucleotide.

**DNA modeling**

DNA 3D models were built with the model.it program as described by Vlahovicek and associates (<http://pongor.itk.ppke.hu/dna/model_it.html>, [Vlahovicek et al, 2003]) [7] from a typical CTS sequence that was predicted to be near the human vascular epidermal growth factor (VEGFA chr17:45,731,352-45,731,368). Furthermore, the curvature/bendability properties were predicted with the bend.it program [7] (<http://pongor.itk.ppke.hu/dna/bend_it.html>). The process used 16964 predicted CTS (identified with ChIA-PET analysis, see above) sequences (all sequences deposited as Table S11 in Additional file 3). The results are shown in Figure S11**.** Figure S11a shows that the DNA double helix is not inherently curved in this region. A consensus prediction of 16964 aligned regions shows that the DNA duplex is slightly more flexible and less curved than the surrounding regions (Figure S11). Mapping the peak shift values in Figures 2a and 2b of the cohesin components onto a circle diagram (representing the top view of the 3D model) shows that CTCF, RAD21 and STAG1/2 map on one face of the helix while SMC1 and SMC3 map on the other face. A side-view of the DNA model shows that the overlapping contact points of SMC1 and SMC3 are in between CTCF and RAD21, on the face opposite to CTCF, RAD21 and STAG1/2 (Figure S11).

**Two case studies: DNA binding analysis of different regulatory complexes**

Our aim was to apply our method to known interactions between protein complexes and the DNA, As such, we examined further DNA binding events with symmetric and asymmetric properties. In this context, asymmetric binding properties resulted in non-zero ChIP-seq peak shifts and that means they can serve as positive controls. On the other hand, no peak shift is expected for symmetrically binding complexes and that means these cases are negative controls

*Positive control*. Forkhead (FOX) proteins have been described as pioneer factors, which, by superseding linker histones, can open up compact DNA [8]. FOX proteins thus are key transcription factors (TFs) in the development of tumorigenesis, e.g. in the steroid hormone dependent cancers. The FOXA1::AR (androgen receptor) composite element has been discovered in human prostate cancer derived cells [9]. In this element there are four spacers between the recognition sites of FOXA1 and AR, thus we would expect a shift between the co-binding proteins on the DNA. We could detect a significant number of sites bound by both FOXA1 and AR in VCaP (3477), and a feasible number of such sites in LNCaP prostate cancer cells (713), where around a 4 bp shift could be observed (at the level of P<2.2x10^-16^ and P=5.06x10^-08^, respectively, according to the Wilcoxon signed-rank test) [10]. This matches well with our preliminary expectations and shows that this amount of ChIP-seq summits is sufficient to determine the strand specific relative location of the co-binding TFs.

*Negative controls.* Conversely, coregulators that do not bind to DNA directly but through TF(s) are expected to show no shift compared to their respective DNA-binding factor(s). Retinoid X receptor (RXR) is the heterodimerizing partner of class II nuclear receptors (NRs), which are activated typically by lipid molecules thus sensing and transducing the environmental signals [11]. For this, they need coregulators such as P300 [12]. Although the NR binding site has direction, one can expect the RXR and P300 at the same location. And indeed, P300 did not show any strand specific shifts relative to the location of RXR at 779 commonly bound NR (half) sites in mouse 3T3-L1 preadipocytes (P=0.5287) [13].

We tested a third complex containing stem cell specific TFs (OCT3/4, SOX2, KLF4 and cMYC), which are probably applicable for the dedifferentiation of any cell types [14]. These complexes also include the NANOG homeodomain protein, which is a pluripotent factor responsible for self-renewal [15]. For the comparison, we used the composite element of OCT3/4 (POU5F1) and SOX2, which showed the co-occurrence of POU5F1 and NANOG proteins at 2206 sites. As NANOG binding – in a similar manner to the coregulators – is secondary, a minimal, statistically insignificant shift (P=0.1776) could be determined compared to the location of POU5F1 in mouse embryonic stem cells [16]

Supplemantary Figures and Tables


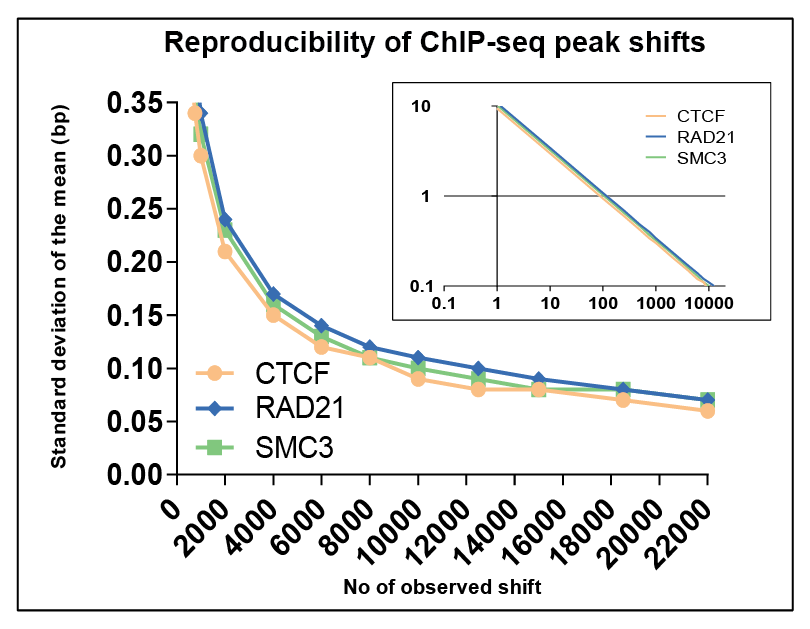


Supplementary Figure S1. The reproducibility of ChIP-seq peak shifts in a HeLa cell experiment.

The reproducibility was characterized as the standard deviation of the mean (Y axis) determined from a number of observed peak shifts (X-axis). The peak shift was measured between the peak summit of the proteins indicated in the figure (CTCF-SRX102984, Rad21-SRX150650, SMC3-SRX150464, as indicated in Table S1 in Additional file 1) and the center of the CTCF binding site (CTS). The inset shows a detail in logarithmic scale that shows that approximately 100 shift values are necessary to reach a reproducibility of +/-1 nucleotide. In our experiments we used typically more than 5000 peaks, which roughly corresponds to a reproducibility of 0.1. These estimates vary with the quality/coverage of the dataset.


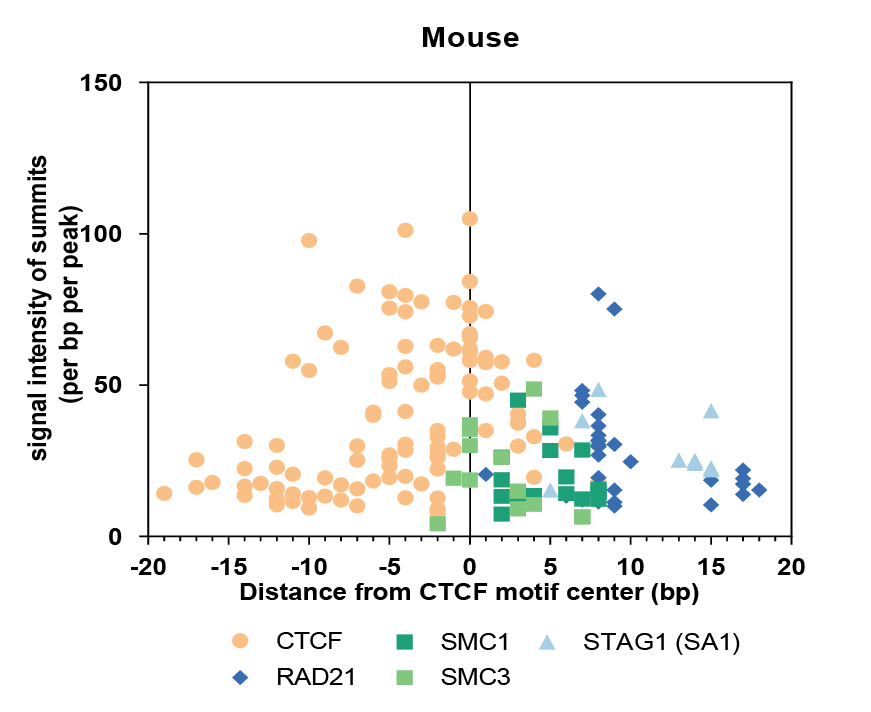


Supplementary Figure S2. Shift between CTCF and cohesin bound sites in mouse cells.

The scatter plot shows the maxima of ChIP fragment coverage of CTCF, RAD21, SMC1/3 and STAG1 on CTSs specific for the given mouse cell or tissue type (see supplementary methods). The vertical axis shows the maxima of the average fragment depth and their position relative to the midpoint of CTSs, which is represented on the horizontal axis. Position weight matrix of the CTCF is shown on the bottom of Fig. 1B.


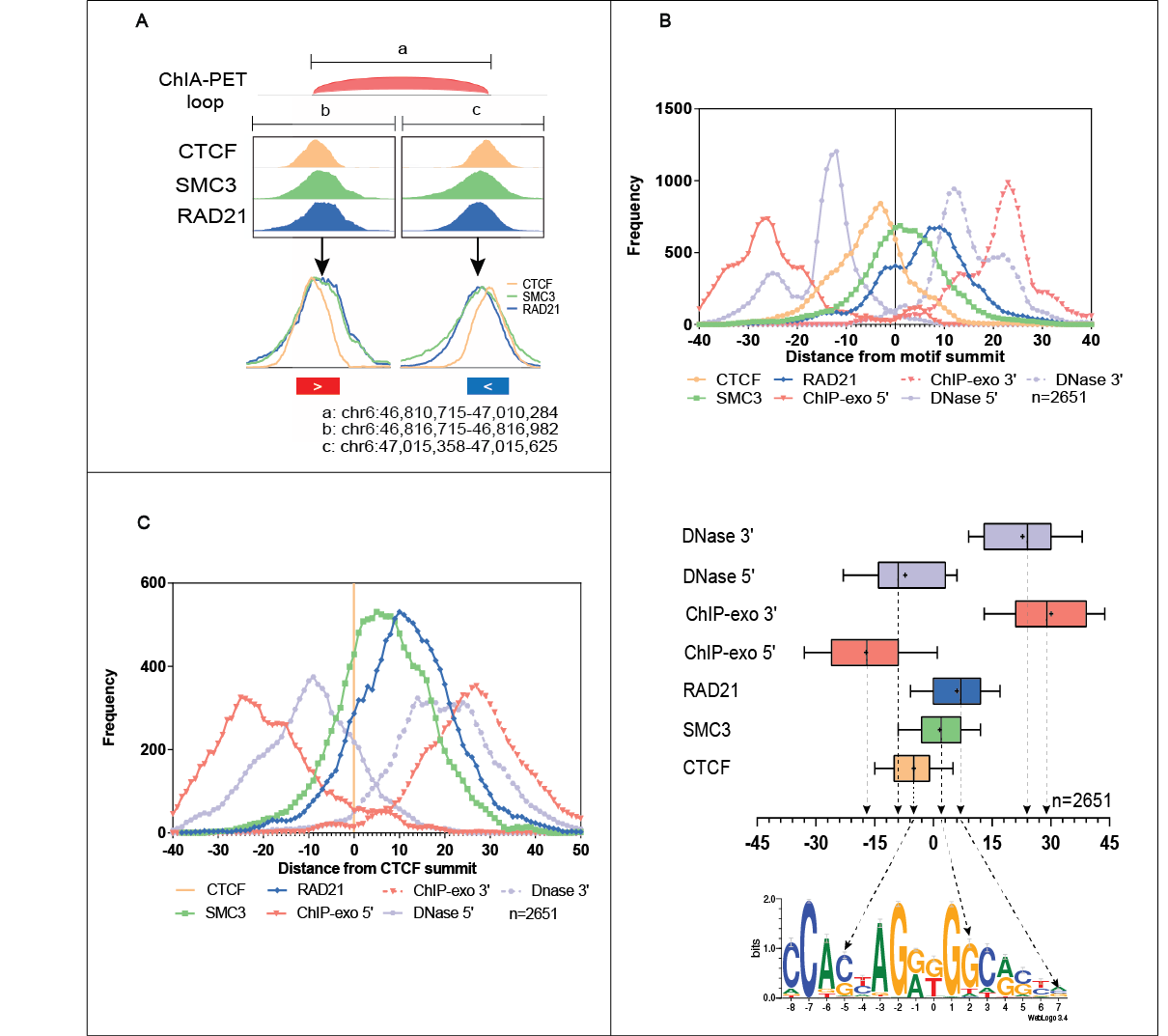


Supplementary Figure S3. The boundaries of genomic regions covered by CTCF/cohesin.

(A) Representative example of the strand specific CTCF-cohesin shift derived from CTCF, SMC3 and RAD21 ChIP-seq data of HeLa. Available ChIA-PET (target: CTCF) was used to identify CTCF binding sites that were involved in chromatin loop (SRX160885). The red and blue boxes indicate the CTCF elements on the forward and reverse strand, respectively.

(B) ChIP-seq peak summit positions of CTCF/cohesin complex components show a conserved, strand-specific distance pattern relative to both the CTS center and the DNase-seq footprint and ChIP-exo borders in HeLa cells (SRA datasets SRX080392, SRX150650, SRX150464, SRX098243, SRX100899). Top: histogram of summit distribution of the CTCF and cohesin bound sites using a 5 bp sliding window. Middle: box plots show the median positions (vertical lines), average positions (“+”), first and third quartiles (box borders) and 10-90 percentiles (whiskers) of the distributions. The bottom panel shows the mapping on the CTCF motif logo.

(C) Distance distribution of cohesin proteins and “edge markers” relative to the CTCF. The horizontal axis represents the distance of peak summits and ChIP-exo and DNase footprint borders relative to the CTCF summits (orange line). The vertical axis represents the distance frequency. Rolling mean with 5 bp window was applied to smooth the frequency.


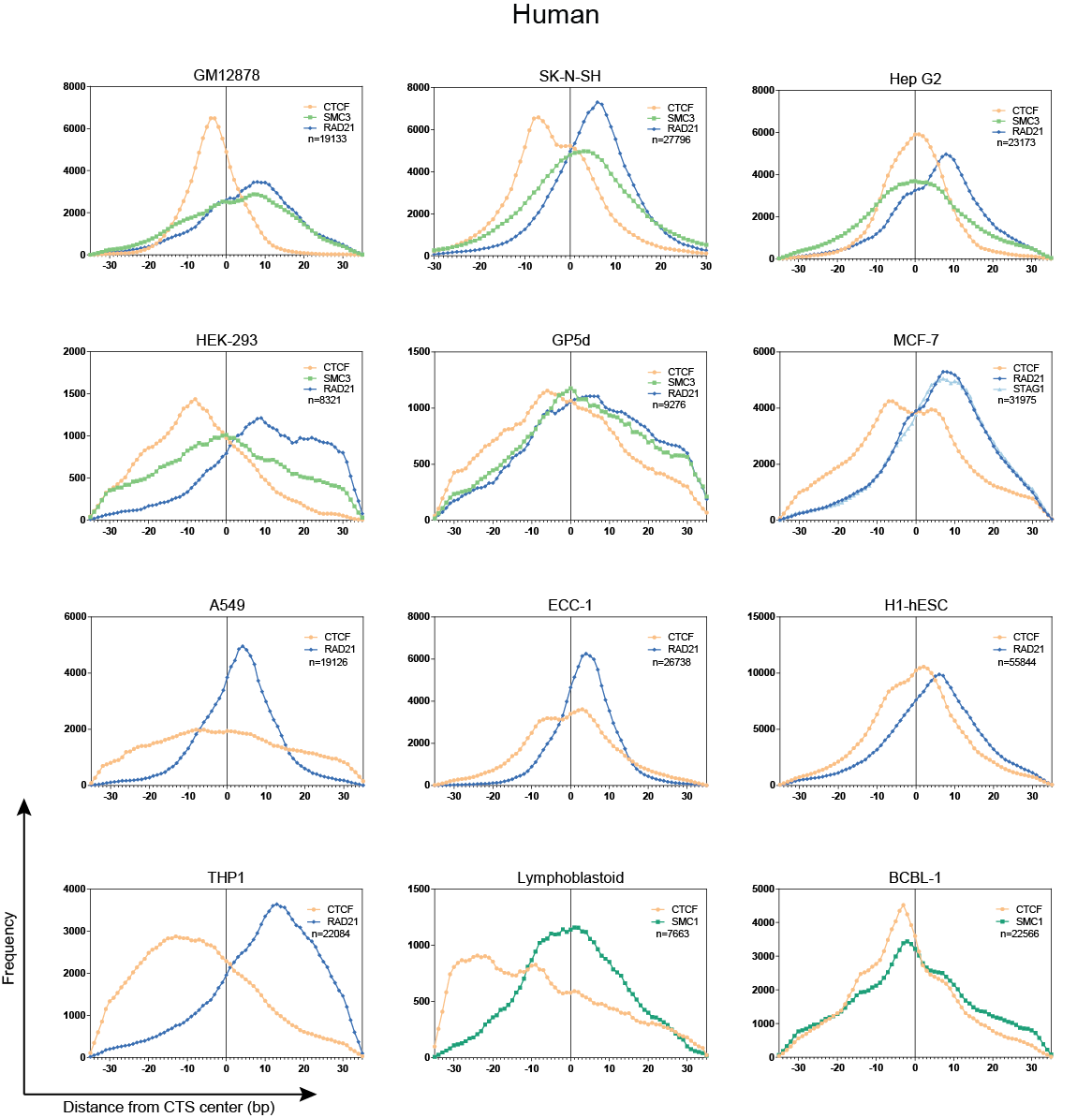


Supplementary Figure S4. Shift between CTCF/cohesin proteins in human cell lines.

Histograms show the distribution of the peak summits of CTCF/cohesin proteins relative to the midpoint of CTSs by using a 5 bp sliding window.


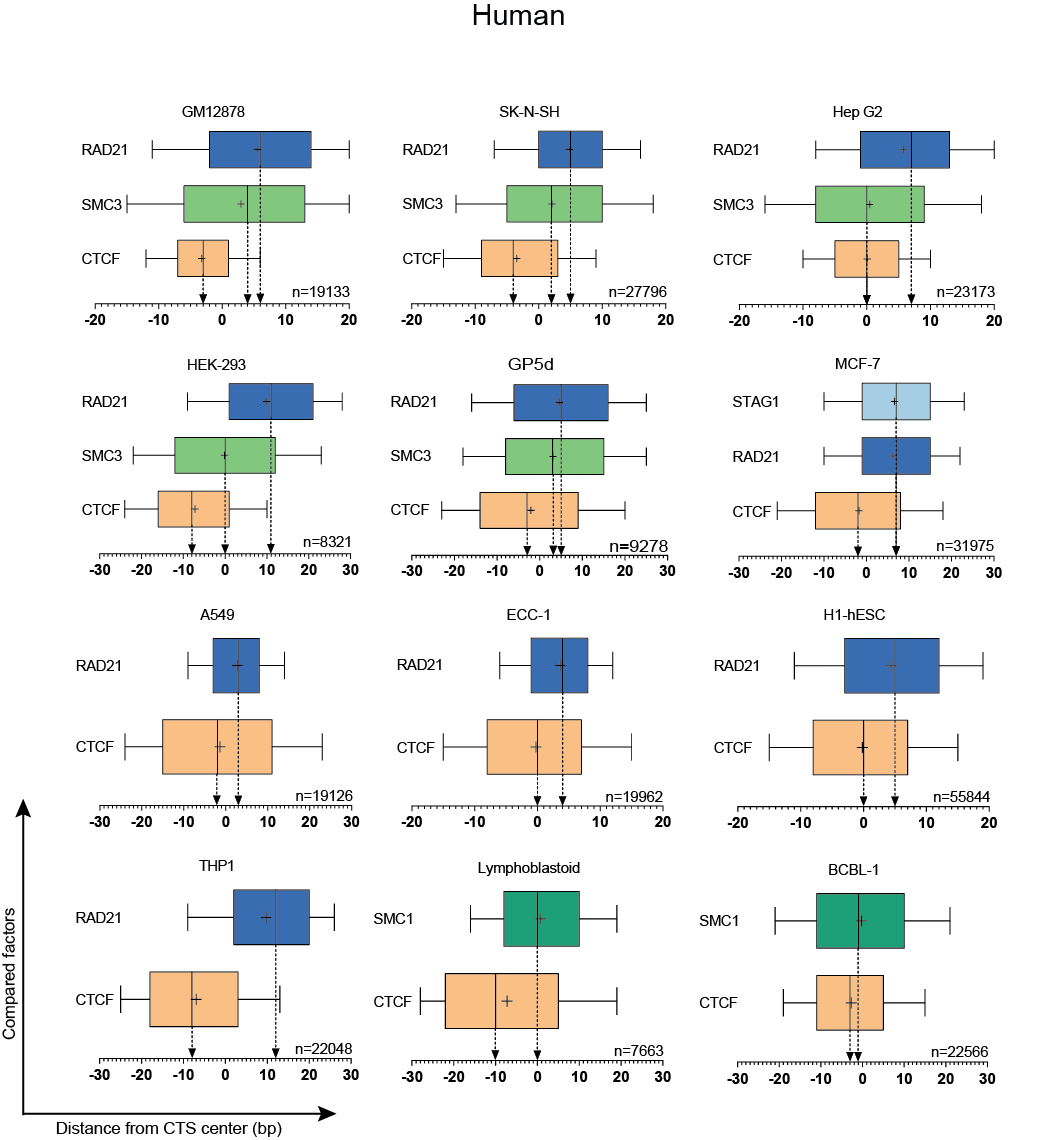


Supplementary Figure S5. Box plot representation of the strand specific shift between CTCF and cohesin proteins in human cell lines.

Box plots show the median positions (vertical lines), average positions (“+”), first and third quartiles (box borders) and 10-90 percentiles (whiskers) of the distributions.

Supplementary Figure S6. Shift between CTCF/cohesin proteins in mouse cell and tissue types.

Histograms show the distribution of the peak summits of CTCF/cohesin proteins relative to the midpoint of CTSs by using a 5 bp sliding window.


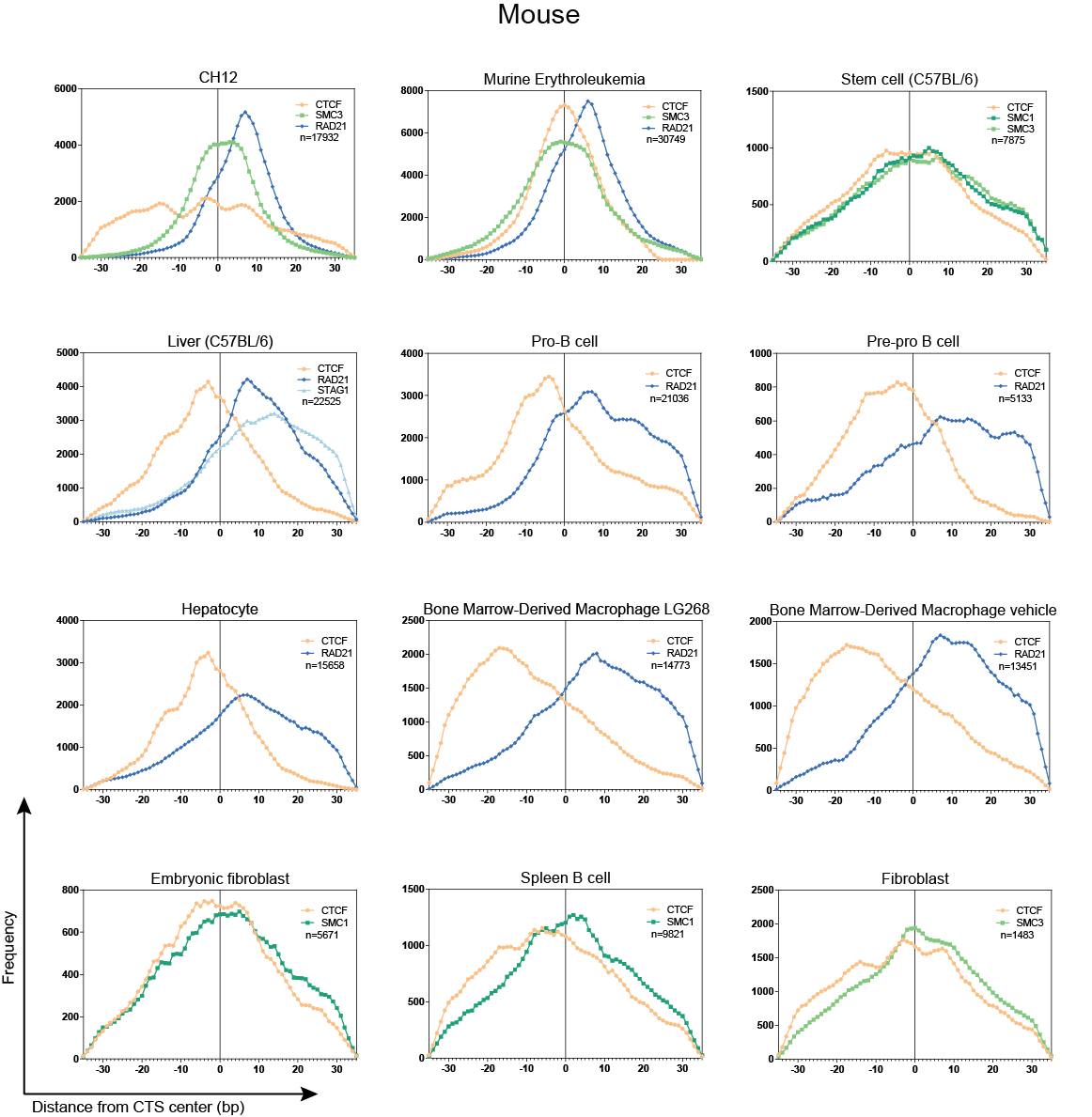


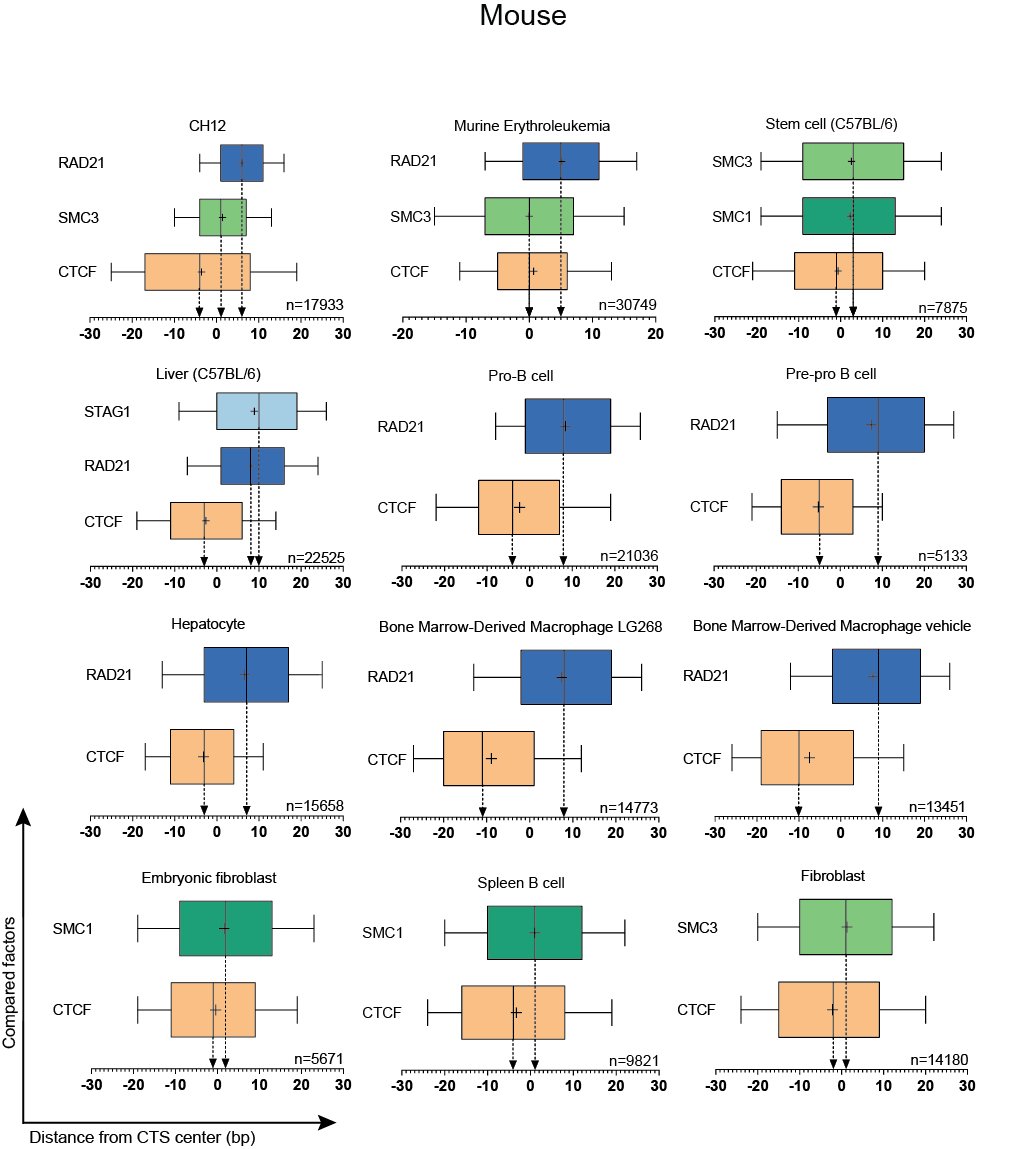


Supplementary Figure S7. Box plot representation of the strand specific shift between CTCF and cohesin proteins in mouse cell and tissue types.

Box plots show the median positions (vertical lines), average positions (“+”), first and third quartiles (box borders) and 10-90 percentiles (whiskers) of the distributions.


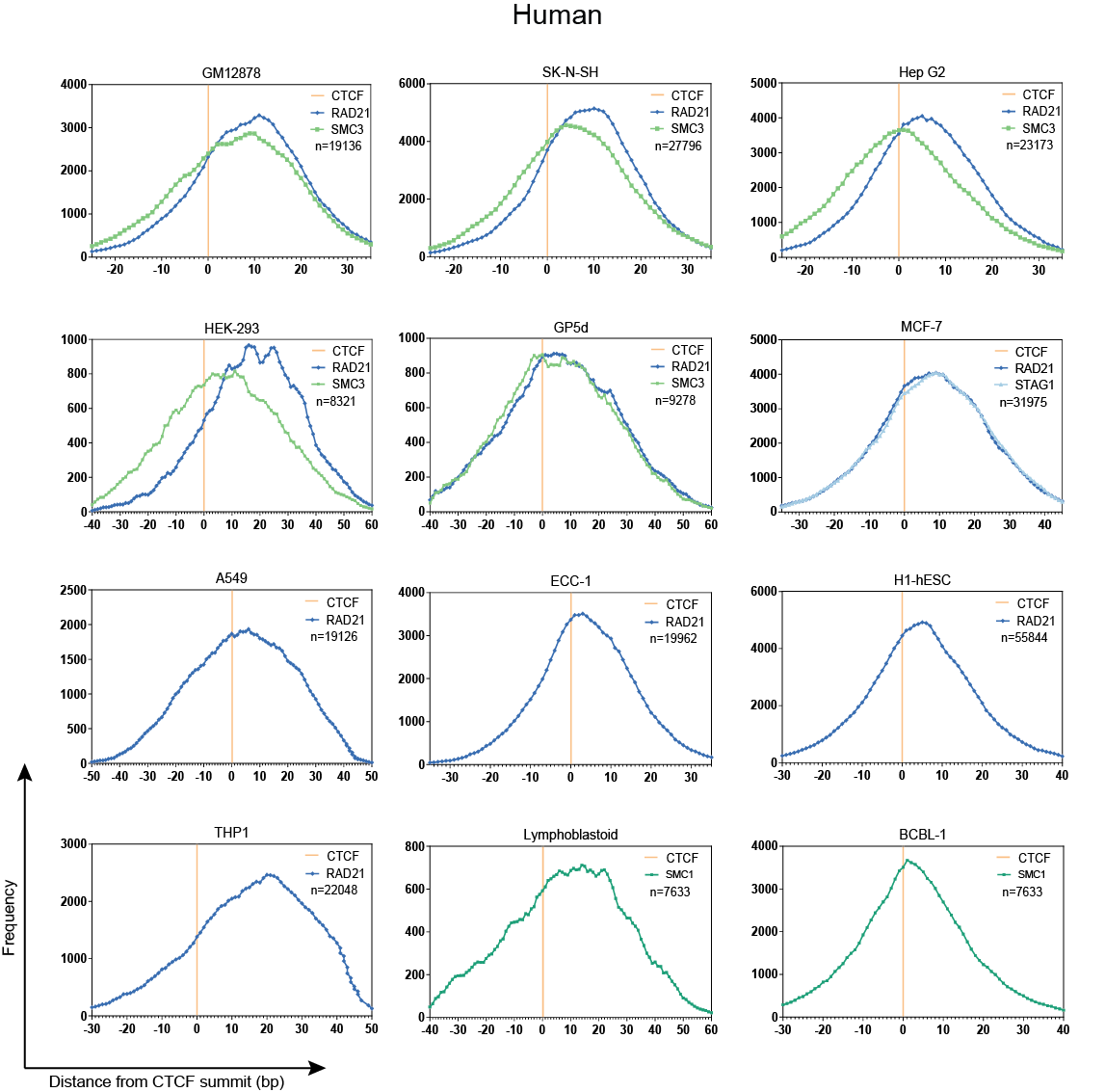


Supplementary Figure S8. Distance distribution of cohesin proteins relative to the CTCF in human cell lines.

Horizontal axis represents the distance of RAD21 (blue curve) and SMC3 summits (green curve) relative to the CTCF summits (orange line) and vertical axis represents the distance frequency. Rolling mean with 5 bp window was applied to smooth the frequency curves.


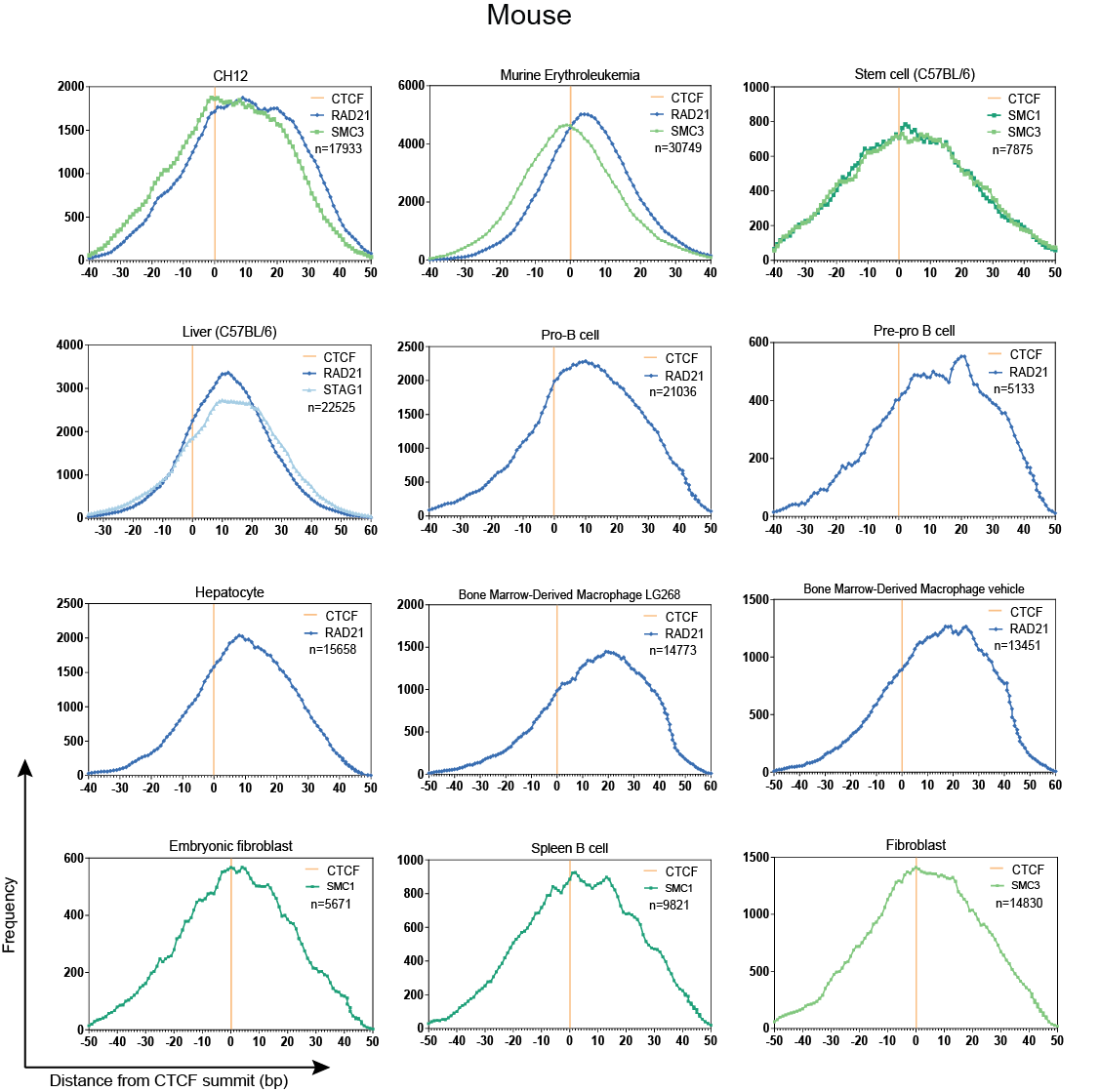


Supplementary Figure S9. Distance distribution of cohesin proteins relative to the CTCF in mouse cell and tissue types.

Horizontal axis represents the distance of RAD21 (blue curve) and SMC3 summits (green curve) relative to the CTCF summits (orange line) and vertical axis represents the distance frequency. Rolling mean with 5 bp window was applied to smooth the frequency curves.


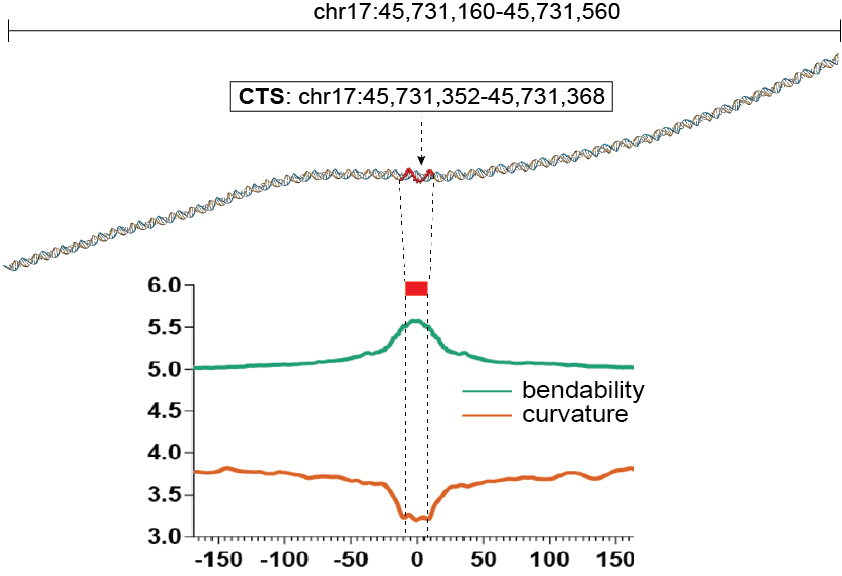


Supplementary Figure S10. DNA modeling.

The model of the CTCF binding site (CTS) and a consensus prediction of 16964 aligned binding sites shows that the DNA double helix is not inherently curved in this region (inset), and that it is slightly less curved and more flexible than the surrounding regions. The model was built with the model.it server (http://pongor.itk.ppke.hu/dna/model_it.html, [18]) from the sequence chr17:45,731,160-45,731,560, the curvature properties in inset were predicted with the bend.it server (http://pongor.itk.ppke.hu/dna/bend_it.html, [18]) using loop involved CTS sequences (all sequences deposited as Table S11 in Additional file 3) [17, 18].


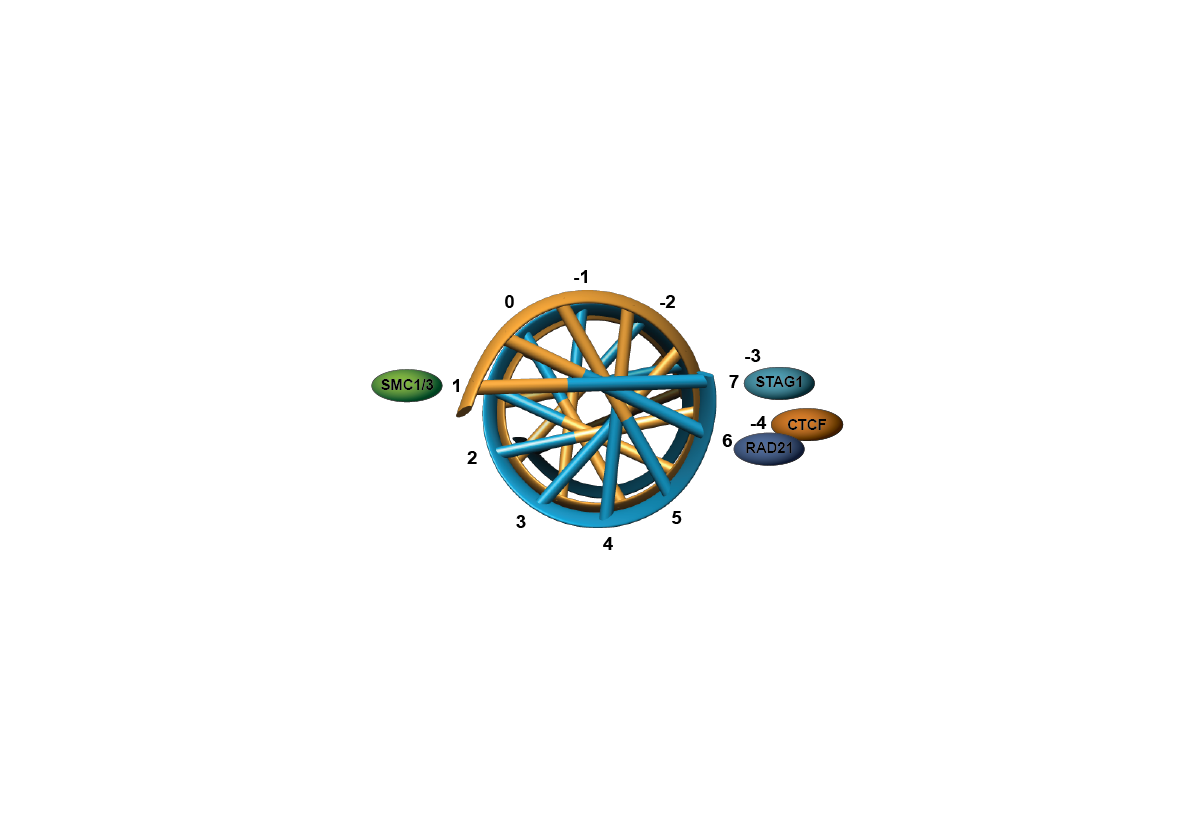


Supplementary Figure S11 Mapping the shift values onto the B-DNA.

A top-view of the DNA model shows that the overlapping contact points of SMC1

and SMC3 are in between CTCF and RAD21, on the opposite face of the helix [17].


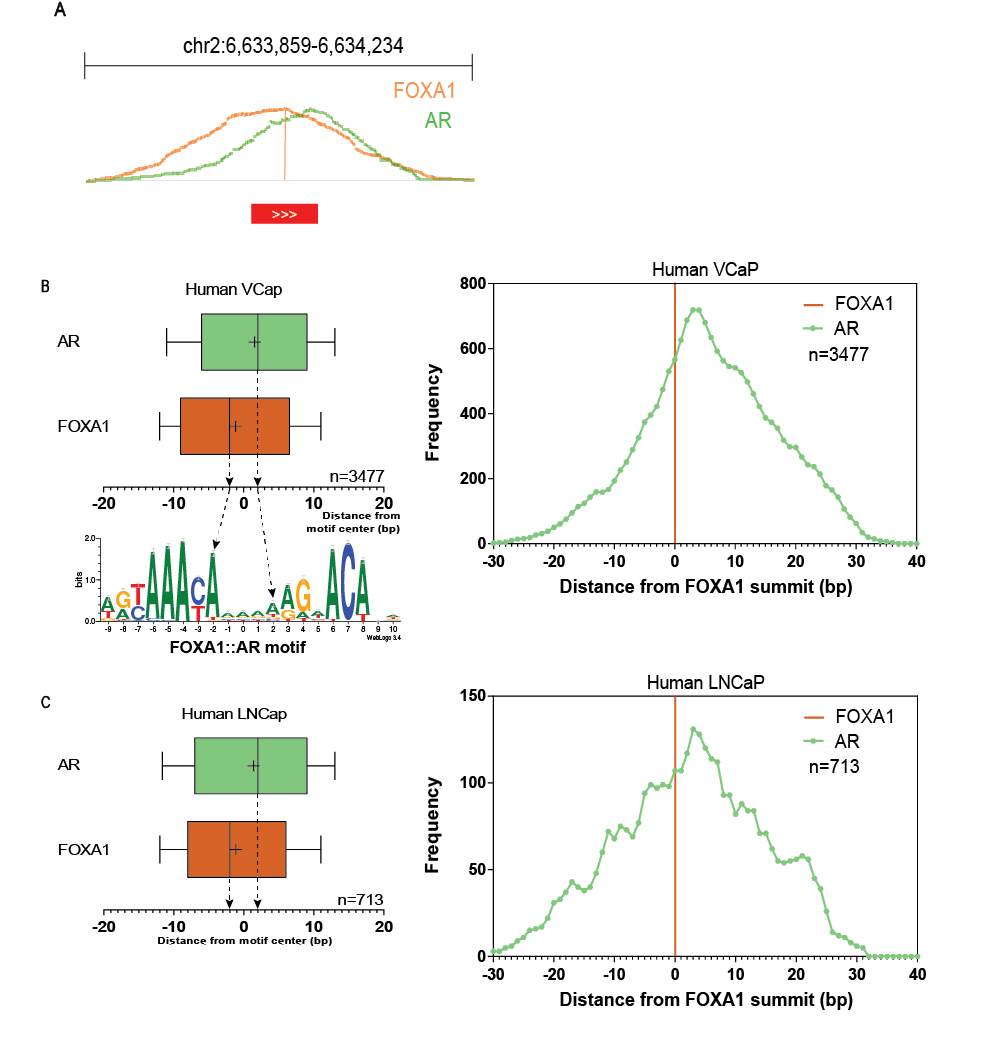
Supplementary Figure S12. Shift between interacting transcription factors (positive control).

(A) Representative example of the strand specific FOXA1/AR shift derived from ChIP-seq data on a human VCaP cell line [19]. The red box indicates the FOXA1::AR composite element.

(B) Box plots indicate the mean (shown as “+”) and median (vertical line) peak summit positions of AR and FOXA1 on 3477 bound regions in VCaP cell line. The bottom panel shows the mapping on the FOXA1:AR motif logo. The histogram (at right) shows the distance distribution of AR relative to the FOXA1 on their common binding sites. The horizontal axis represents the distance of AR summits (green curve) relative to the FOXA1 summits (orange line) while the vertical axis represents the distance frequency. A rolling mean with 5 bp window was applied to smooth the frequency curves.

(C) The relationship of FOXA1 and AR on their 713 composite elements (shown in Figure S12B) in LNCaP cell line.


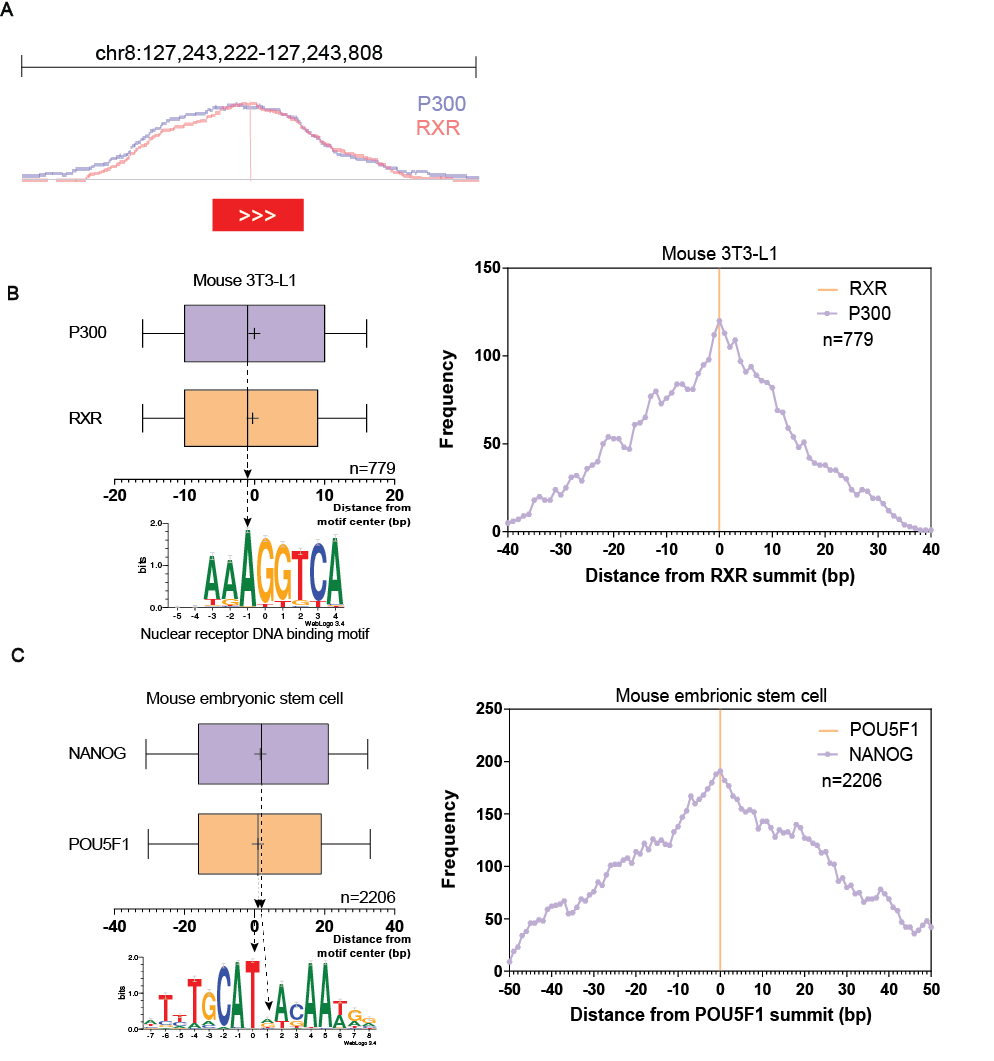


Supplementary Figure S13. Lack of shift between interacting transcriptional regulator proteins (negative control)

(A) Representative example of the lack of the strand specific RXR/P300 shift derived from mouse 3T3-L1 cell line [13]. The red box indicates the nuclear receptor half site.

(B) Box plots indicate the mean (shown as “+”) and median (vertical line) peak summit positions of P300 and RXR on 779 bound regions in 3T3-L1 cell line. The bottom panel shows the mapping on the nuclear receptor motif logo. The histogram (at right) shows the distance distribution of P300 relative to the RXR on the commonly occupied regions. The horizontal axis represents the distance of P300 summits (purple curve) relative to the RXR summits (orange line) and the vertical axis represents the distance frequency. A rolling mean with 5 bp window was applied to smooth the frequency curves.

(C) The relationship of NANOG and POU5F1 on 2206 POU5F1 binding site in mouse embryonic stem cell [16].

| Step | Task | Program/Package | settings | Remarks |
| --- | --- | --- | --- | --- |
| 1 | Read alignment | BWA version 0.7.10-r789 (4) | –B 8 |  |
| 2 | Peak Identification | MACS2 (5) | -b - SPMR | [Peaks in the ENCODE (2) blacklisted regions removed](file:///C:\Users\Erik\Desktop\supplementary_table_v1.xlsx#RANGE!_ENREF_3) |
| 3 | Peak summit identification | MACS2 (5) |  |  |
| 3 | Motif Enrichment | HOMER (6) | findMotifsGenome.pl -mask -len 10, 12, 14, 16 -dumpFasta -bits -preparse -homer2 -size 100 | Using the top 5000 MACS peaks |
| 4 | Motif remap | HOMER (6) | annotatePeaks.pl  -noann -nogene  -mbed -m | Using the top 5000 MACS peaks |
| 6 | Data visualization | HOMER (6) | makeUCSCfile | For IGV genome viewer (6,7) |

Supplementary Table S3. Steps of ChIP-seq analysis pipeline.

The table summarizes the main steps and programs which were used in raw data processing.

|  | | Cell line | Factor 1 | Factor2 | Wilcoxon signed rank p-value |
| --- | --- | --- | --- | --- | --- |
| Negative control | | 3T3L1 | RXR | P300 | 0.5287 |
|  |  | Stem cell | POU5F1 | NANOG | 0.1776 |
|  |  |  |  |  |  |
| Positive control | | LNCap | FOXA1 | AR | 5.06x10^-8^ |
|  |  | VCap | FOXA1 | AR | < 2.2x10^-16^ |
|  |  |  |  |  |  |
| SAMPLE PAIRS | HUMAN | A549 | CTCF | RAD21 | < 2.2x10^-16^ |
|  |  | ECC-1 | CTCF | RAD21 | < 2.2x10^-16^ |
|  |  | H1-hESC | CTCF | RAD21 | < 2.2x10^-16^ |
|  |  | THP1 | CTCF | RAD21 | < 2.2x10^-16^ |
|  |  | Lymphoblastoid | CTCF | SMC1 | < 2.2x10^-16^ |
|  |  | BCBL-1 | CTCF | SMC1 | < 2.2x10^-16^ |
|  |  |  |  |  |  |
|  | MOUSE | Pro-B cell | CTCF | RAD21 | < 2.2x10^-16^ |
|  |  | Pre-pro B cell | CTCF | RAD21 | < 2.2x10^-16^ |
|  |  | Hepatocyte | CTCF | RAD21 | < 2.2x10^-16^ |
|  |  | BMDM LG26 | CTCF | RAD21 | < 2.2x10^-16^ |
|  |  | BMDM vehicle | CTCF | RAD21 | < 2.2x10^-16^ |
|  |  | Embryonic fibroblast | CTCF | SMC1 | < 2.2x10^-16^ |
|  |  | Spleen B cell | CTCF | SMC1 | < 2.2x10^-16^ |
|  |  | Fibroblast | CTCF | SMC3 | < 2.2x10^-16^ |

Supplementary Table S4. Results of statistic analysis in case of two coherent samples.

Wilcoxon signed-rank test was used in the statistical analysis comparing two matched samples.

|  | Cell Line | Factor 1 | Factor 2 | Friedman Test p value | Namenyi post-hoc test p-values | | |
| --- | --- | --- | --- | --- | --- | --- | --- |
|  |  |  |  |  | CTCF vs Factor1 | CTCF vs Factor2 | Factor1 vs Factor 2 |
| HUMAN | GM12878 | SMC3 | RAD21 | < 2.2x10^-16^ | < 2.0x10^-16^ | < 2.0x10^-16^ | < 2.0x10^-16^ |
|  | GP5d | SMC3 | RAD21 | 1.37x10^-06^ | 3.7x10^-6^ | 0.00025 | 0.62614 |
|  | HEK-293 | SMC3 | RAD21 | < 2.2x10^-16^ | < 2.0x10^-16^ | < 2.0x10^-16^ | < 2.0x10^-16^ |
|  | HeLa | SMC3 | RAD21 | < 2.2x10^-16^ | < 2.0x10^-16^ | < 2.0x10^-16^ | < 2.0x10^-16^ |
|  | Hep G2 | SMC3 | RAD21 | < 2.2x10^-16^ | 1.1x10^-1^ | < 2.0x10^-16^ | < 2.0x10^-16^ |
|  | MCF7 | RAD21 | STAG1 | < 2.2x10^-16^ | < 2.0x10^-16^ | < 2.0x10^-16^ | 0.16 |
|  | SK-N-SH | SMC3 | RAD21 | < 2.2x10^-16^ | < 2.0x10^-16^ | < 2.0x10^-16^ | < 2.0x10^-16^ |
|  |  |  |  |  |  |  |  |
| MOUSE | Ch12 | SMC3 | RAD21 | < 2.2x10^-16^ | < 2.0x10^-16^ | < 2.0x10^-16^ | < 2.0x10^-16^ |
|  | Liver (C57BL/6) | RAD21 | STAG1 | < 2.2x10^-16^ | < 2.0x10^-16^ | < 2.0x10^-16^ | 2.0x10^-11^ |
|  | MEL | RAD21 | SMC3 | 2.0x10^-14^ | < 2.0x10^-16^ | < 2.0x10^-16^ | < 2.0x10^-16^ |
|  | Stem cell (C57BL/6) | SMC1 | SMC3 | < 2.2x10^-16^ | 1.2x10^-14^ | < 2.0x10^-16^ | 0.87 |

Supplementary Table S5. Results of statistic analysis in case of more then two coherent samples.

We used Freidman test with Namenyi post-hoc test in the case of CTCF samples that had more than two available parallel cohesin ChIP-seq data sets.

|  | Cell line | Cohesin 1. | Cohesin 2. | Median position | | | Mean position | | | motif number |
| --- | --- | --- | --- | --- | --- | --- | --- | --- | --- | --- |
|  |  |  |  | CTCF | Cohesin 1. | Cohesin 2. | CTCF | Cohesin 1. | Cohesin 2. |  |
| HUMAN | A549 | RAD21 | - | -2 | 3 | - | -1.32 | 2.78 | - | 19126 |
|  | BCBL-1 | SMC1 | - | -3 | -1 | - | -2.70 | -0.26 | - | 22566 |
|  | ECC-1 | RAD21 | - | 0 | 4 | - | -0.26 | 3.65 | - | 26738 |
|  | GM12878 | SMC3 | RAD21 | -3 | 4 | 6 | -3.22 | 2.98 | 5.54 | 19133 |
|  | GP5d | SMC3 | RAD21 | -3 | 3 | 5 | -2.04 | 3.13 | 4.55 | 9276 |
|  | H1-hESC | RAD21 | - | 0 | 5 | - | -0.19 | 4.37 | - | 55844 |
|  | HEK-293 | SMC3 | RAD21 | -8 | 0 | 11 | -7.20 | -0.11 | 9.97 | 8321 |
|  | HeLa | SMC3 | RAD21 | -4 | 1 | 6 | -4.61 | 1.50 | 5.22 | 21994 |
|  | HepG2 | SMC3 | RAD21 | 0 | 0 | 7 | 0.09 | 0.47 | 5.80 | 23173 |
|  | Lymphoblastoid | SMC1 | - | -10 | 0 | - | -7.24 | 0.80 | - | 7663 |
|  | MCF-7 | RAD21 | STAG1 (SA1) | -2 | 7 | 7 | -1.78 | 6.26 | 6.57 | 31975 |
|  | SK-N-SH | SMC3 | RAD21 | -4 | 2 | 5 | -3.44 | 2.10 | 4.83 | 27796 |
|  | THP1 | RAD21 | - | -8 | 12 | - | -6.95 | 9.75 | - | 22048 |
|  |  |  |  |  |  |  |  |  |  |  |
| MOUSE | CH12 | SMC3 | RAD21 | -4 | 1 | 6 | -3.59 | 1.41 | 6.02 | 17932 |
|  | Embryonic fibroblast | SMC1 | - | -1 | 2 | - | -0.42 | 1.65 | - | 5671 |
|  | Fibroblast | SMC3 | - | -2 | 1 | - | -2.16 | 1.29 | - | 14830 |
|  | Hepatocyte | RAD21 | - | -3 | 7 | - | -3.16 | 6.63 | - | 15658 |
|  | Liver (C57BL/6) | RAD21 | STAG1 (SA1) | -3 | 9 | 11 | -2.98 | 8.81 | 9.87 | 22525 |
|  | MEL | SMC3 | RAD21 | 0 | 0 | 5 | 0.70 | -0.01 | 5.17 | 30749 |
|  | Pro-B cell | RAD21 | - | -4 | 8 | - | -2.33 | 8.44 | - | 21036 |
|  | Pre-pro B cell | RAD21 | - | -5 | 9 | - | -5.21 | 7.38 | - | 5133 |
|  | Spleen B cell | SMC1 | - | -4 | 1 | - | -3.28 | 1.01 | - | 9821 |
|  | Stem cell (C57BL/6) | SMC1 | SMC3 | -1 | 3 | 3 | -0.60 | 2.28 | 2.61 | 7875 |
|  | BMDM LG268 | RAD21 | - | -11 | 8 | - | -8.90 | 7.47 | - | 14773 |
|  | BMDM vehicle | RAD21 | - | -10 | 9 | - | -7.44 | 7.69 | - | 13451 |

Supplementary Table S6. Summary table of CTCF-cohesin samples.

The table summerize the average and median positions of factor summits relative to the center of the CTS.

|  |  | Mean | | | |  | Median | | | |
| --- | --- | --- | --- | --- | --- | --- | --- | --- | --- | --- |
|  |  | CTCF | RAD21 | SMC1/3 | STAG1 |  | CTCF | RAD21 | SMC1/3 | STAG1 |
| SCATTER PLOT VALUES | MOUSE | -4.29 | 9.47 | 3.63 | 12.00 |  | -4 | 8 | 3 | 14 |
|  | HUMAN | -3.83 | 6.00 | 0.63 | 7.33 |  | -4 | 5 | 0.5 | 8 |
|  | HUMAN + MOUSE | -3.99 | 7.89 | 3.02 | 11.06 |  | -4 | 8 | 3 | 13 |
|  |  |  |  |  |  |  |  |  |  |  |
| BOX PLOT VALUES | MOUSE | -3.00 | 7.07 | 1.07 | 9.87 |  | -3 | 7 | 1 | 11 |
|  | HUMAN | -2.38 | 4.97 | 1.34 | 6.57 |  | -3 | 6 | 1 | 7 |
|  | HUMAN + MOUSE | -2.62 | 5.70 | 1.23 | 7.93 |  | -3 | 6 | 1 | 9 |

Supplementary Table S7. Average values of CTCF/cohesin proteins related to CTS.

The table shows the calculated mean and median position of the samples.

|  | Cell line | Cohesin 1. | Cohesin 2. | Median distance | |  | Mean distance | | motif number |
| --- | --- | --- | --- | --- | --- | --- | --- | --- | --- |
|  |  |  |  | Cohesin 1 - CTCF | Cohesin 2 - CTCF |  | Cohesin 1 - CTCF | Cohesin 2 - CTCF |  |
| HUMAN | A549 | RAD21 | - | 4 | - |  | 4.10 | - | 19126 |
|  | BCBL-1 | SMC1 | - | 2 | - |  | 2.44 | - | 22566 |
|  | ECC-1 | RAD21 | - | 4 | 0 |  | 3.82 | - | 26738 |
|  | GM12878 | SMC3 | RAD21 | 7 | 9 |  | 6.20 | 8.76 | 19133 |
|  | GP5d | SMC3 | RAD21 | 5 | 7 |  | 5.17 | 6.60 | 9276 |
|  | H1-hESC | RAD21 | - | 5 | - |  | 4.56 | - | 55844 |
|  | HEK-293 | SMC3 | RAD21 | 7 | 18 |  | 7.09 | 17.17 | 8321 |
|  | HeLa | SMC3 | RAD21 | 6 | 10 |  | 6.11 | 9.83 | 21991 |
|  | HepG2 | SMC3 | RAD21 | 0 | 6 |  | 0.38 | 5.70 | 23173 |
|  | Lymphoblastoid | SMC1 | - | 10 | - |  | 8.04 | - | 7663 |
|  | MCF-7 | RAD21 | STAG1 (SA1) | 8 | 9 |  | 8.04 | 8.35 | 31975 |
|  | SK-N-SH | SMC3 | RAD21 | 6 | 8 |  | 5.55 | 8.28 | 27796 |
|  | THP1 | RAD21 | - | 18 | 8 |  | 16.71 | - | 22048 |
|  |  |  |  |  |  |  |  |  |  |
| MOUSE | CH12 | SMC3 | RAD21 | 5 | 10 |  | 5.00 | 9.61 | 17932 |
|  | Embryonic fibroblast | SMC1 | - | 2 | - |  | 2.07 | - | 5671 |
|  | Fibroblast | SMC3 | - | 4 | - |  | 3.45 | - | 14830 |
|  | Hepatocyte | RAD21 | - | 10 | 3 |  | 9.79 | 3.16 | 15658 |
|  | Liver (C57BL/6) | RAD21 | STAG1 (SA1) | 12 | 13 |  | 11.79 | 12.85 | 22525 |
|  | MEL | SMC3 | RAD21 | -1 | 5 |  | -0.71 | 4.47 | 30749 |
|  | Pro-B cell | RAD21 | - | 11 | - |  | 10.77 | - | 21036 |
|  | Pre-pro B cell | RAD21 | - | 13 | - |  | 12.59 | - | 5133 |
|  | Spleen B cell | SMC1 | - | 4 | - |  | 4.29 | - | 9821 |
|  | Stem cell (C57BL/6) | SMC1 | SMC3 | 3 | 3 |  | 2.88 | 3.20 | 7875 |
|  | BMDM LG268 | RAD21 | - | 18 | - |  | 16.37 | - | 14773 |
|  | BMDM vehicle | RAD21 | - | 16 | - |  | 15.13 | - | 13451 |

Supplementary Table S8. Median and Mean distance from CTCF summits.

The table shows the median and average distances of cohesin subunits from CTCF summits.


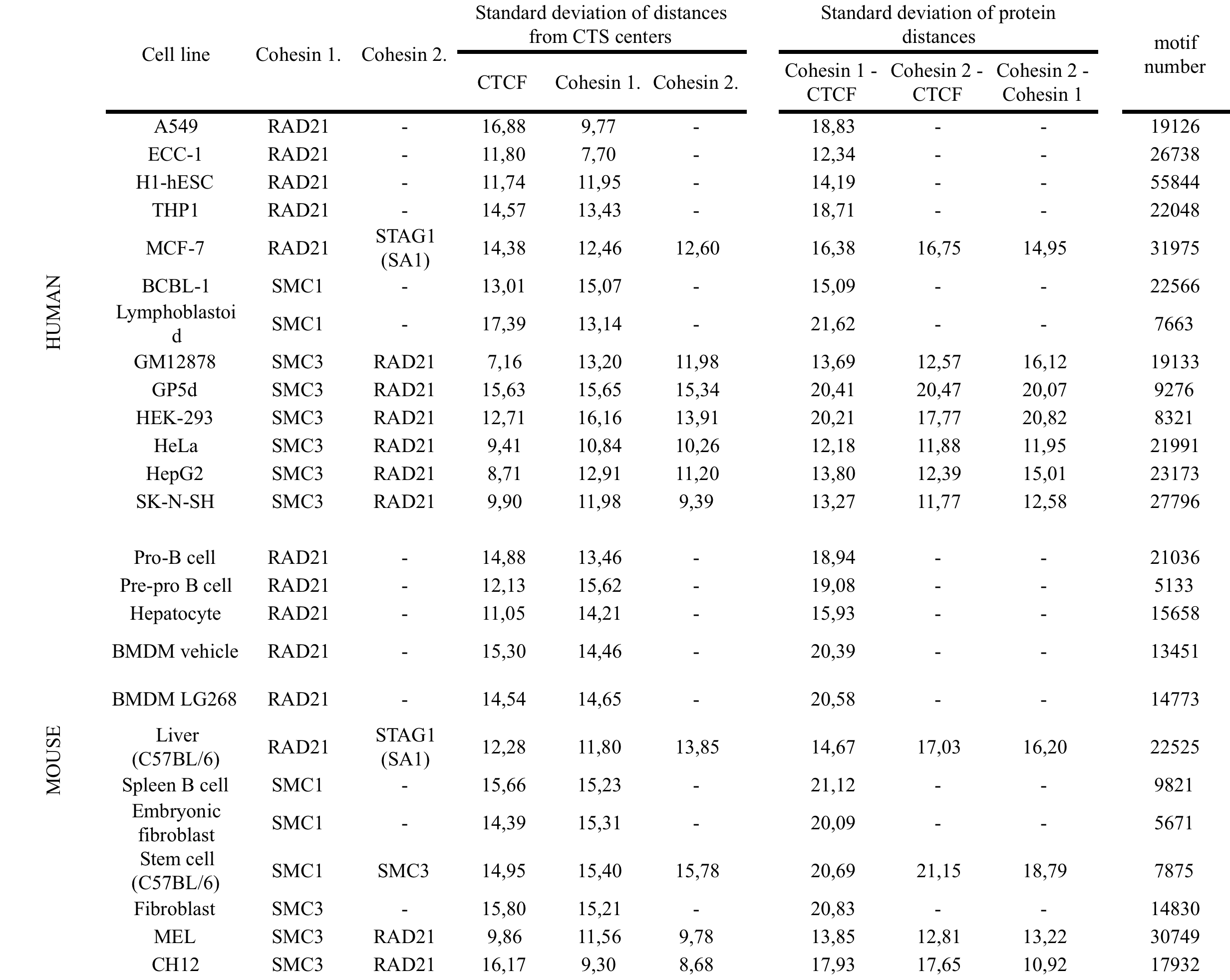


Supplementary Table S9. Standard deviation of protein distances near CTSs

|  |  |  | Median | | |  | Mean | | | Population number |
| --- | --- | --- | --- | --- | --- | --- | --- | --- | --- | --- |
|  |  |  | Position | | Factor 2 - Factor 1 distance |  | Position | | Factor 2 - Factor 1 distance |  |
|  | Factor 1. | Factor 2. | Factor 1. | Factor 2. |  |  | Factor 1. | Factor 2. |  |  |
| Human Vcap | FOXA1 | AR | -2 | 2 | 2 |  | -1.13661 | 1.59218 | 2.72879 | 3477 |
| Human LNCap | FOXA1 | AR | -2 | 2 | 3 |  | -1.09818 | 1.42777 | 2.52595 | 713 |
| Mouse 3T3-L1 | RXR | P300 | 1 | 2 | 1 |  | 0.99093 | 1.64778 | 0.65684 | 779 |
| Mouse embryonic stem cell | POU5F1 | NANOG | -1 | -1 | 1 |  | -0.27728 | -0.0193 | 0.25802 | 2206 |

Supplementary Table S10. Relative positions of the co-occupied transcription regulators used as controls.

The table summarizes the boxplot data of controls. Factor1 and Factor2 are those transcription regulator proteins whose relative positions are compared to each other. Mean and median distances are shown relative to their reference points. Each pair has their own reference point, which is the midpoint of a suitable motif. The motifs are shown with boxplot figures. The population number represents the size of the consensus motif set. The consensus motif set incorporate those motif regions that can be found exactly on the same genomic position in both members of the pair.

Additional Data Table S1. Basic information of human samples.

The table summarizes the data of quality and availability of samples. The table includes the maxima positions related to the CTSs. (data shown on scatterplot Figure 1A)

Additional Data Table S2. Basic information of mouse samples.

The table summarizes the data of quality and availability of samples. The table includes the maxima positions related to the CTSs. (data shown on scatterplot Figure S2.)

Additional Data Table S11. Investigation of curvature/bendability on CTS-centered sequences.

The table shows the sequence of 400 bp frame of 16964 CTSs, which were identified with ChIA-PET analysis sequences.

References:

1. R Development Core Team. R: A language and environment for statistical computing. Vienna, Austria: R Foundation for Statistical Computing 2014.

2. Wickham H. Reshaping Data with the reshape Package. 2007. 2007;21(12):20. doi:10.18637/jss.v021.i12.

3. Pohlert T. The Pairwise Multiple Comparison of Mean Ranks Package (PMCMR). 2014.

4. Heinz S, Benner C, Spann N, Bertolino E, Lin YC, Laslo P et al. Simple combinations of lineage-determining transcription factors prime cis-regulatory elements required for macrophage and B cell identities. Molecular cell. 2010;38(4):576-89. doi:10.1016/j.molcel.2010.05.004.

5. Quinlan AR, Hall IM. BEDTools: a flexible suite of utilities for comparing genomic features. Bioinformatics (Oxford, England). 2010;26(6):841-2. doi:10.1093/bioinformatics/btq033.

6. Robinson JT, Thorvaldsdóttir H, Winckler W, Guttman M, Lander ES, Getz G et al. Integrative Genomics Viewer. Nature Biotechnology. 2011;29(1):24-6. doi:10.1038/nbt.1754.

7. Vlahoviček K, Kaján L, Murvai J, Hegedűs Z, Pongor S. The SBASE domain sequence library, release 10: domain architecture prediction. Nucleic acids research. 2003;31(1):403-5.

8. Cirillo LA, McPherson CE, Bossard P, Stevens K, Cherian S, Shim EY et al. Binding of the winged-helix transcription factor HNF3 to a linker histone site on the nucleosome. Embo j. 1998;17(1):244-54. doi:10.1093/emboj/17.1.244.

9. Sahu B, Laakso M, Ovaska K, Mirtti T, Lundin J, Rannikko A et al. Dual role of FoxA1 in androgen receptor binding to chromatin, androgen signalling and prostate cancer. Embo j. 2011;30(19):3962-76. doi:10.1038/emboj.2011.328.

10. Toropainen S, Malinen M, Kaikkonen S, Rytinki M, Jaaskelainen T, Sahu B et al. SUMO ligase PIAS1 functions as a target gene selective androgen receptor coregulator on prostate cancer cell chromatin. Nucleic Acids Res. 2015;43(2):848-61. doi:10.1093/nar/gku1375.

11. Mangelsdorf DJ, Thummel C, Beato M, Herrlich P, Schutz G, Umesono K et al. The nuclear receptor superfamily: the second decade. Cell. 1995;83(6):835-9.

12. Daniel B, Nagy G, Hah N, Horvath A, Czimmerer Z, Poliska S et al. The active enhancer network operated by liganded RXR supports angiogenic activity in macrophages. Genes Dev. 2014;28(14):1562-77. doi:10.1101/gad.242685.114.

13. Siersbaek R, Rabiee A, Nielsen R, Sidoli S, Traynor S, Loft A et al. Transcription factor cooperativity in early adipogenic hotspots and super-enhancers. Cell Rep. 2014;7(5):1443-55. doi:10.1016/j.celrep.2014.04.042.

14. Takahashi K, Yamanaka S. Induction of pluripotent stem cells from mouse embryonic and adult fibroblast cultures by defined factors. Cell. 2006;126(4):663-76. doi:10.1016/j.cell.2006.07.024.

15. Chambers I, Colby D, Robertson M, Nichols J, Lee S, Tweedie S et al. Functional expression cloning of Nanog, a pluripotency sustaining factor in embryonic stem cells. Cell. 2003;113(5):643-55.

16. Galonska C, Ziller MJ, Karnik R, Meissner A. Ground State Conditions Induce Rapid Reorganization of Core Pluripotency Factor Binding before Global Epigenetic Reprogramming. Cell stem cell. 2015;17(4):462-70. doi:10.1016/j.stem.2015.07.005.

17. Pettersen EF, Goddard TD, Huang CC, Couch GS, Greenblatt DM, Meng EC et al. UCSF Chimera--a visualization system for exploratory research and analysis. Journal of computational chemistry. 2004;25(13):1605-12. doi:10.1002/jcc.20084.

18. Vlahovicek K, Kajan L, Pongor S. DNA analysis servers: plot.it, bend.it, model.it and IS. Nucleic Acids Res. 2003;31(13):3686-7.

19. Toropainen S, Malinen M, Kaikkonen S, Rytinki M, Jääskeläinen T, Sahu B et al. SUMO ligase PIAS1 functions as a target gene selective androgen receptor coregulator on prostate cancer cell chromatin. Nucleic acids research. 2015;43(2):848-61. doi:10.1093/nar/gku1375.
